# Contextual perception under active inference

**DOI:** 10.1038/s41598-021-95510-9

**Published:** 2021-08-10

**Authors:** M. Berk Mirza, Maell Cullen, Thomas Parr, Sukhi Shergill, Rosalyn J. Moran

**Affiliations:** 1 Centre for Neuroimaging Sciences, Department of Neuroimaging, Institute of Psychiatry, Psychology and Neuroscience, King’s College London, London, UK; 2 The NIHR Maudsley Biomedical Research Centre (BRC) at South London and Maudsley NHS Foundation Trust and the Institute of Psychiatry, Psychology and Neuroscience, King’s College London, London, UK; 3 Department of Engineering Mathematics, University of Bristol, Bristol, UK; 4 Wellcome Centre for Human Neuroimaging, Institute of Neurology, University College London, London, UK; 5 Department of Psychosis Studies, Institute of Psychiatry, Psychology and Neuroscience, King’s College London, London, UK

**Keywords:** Cognitive neuroscience, Computational neuroscience, Emotion

## Abstract

Human social interactions depend on the ability to resolve uncertainty about the mental states of others. The context in which social interactions take place is crucial for mental state attribution as sensory inputs may be perceived differently depending on the context. In this paper, we introduce a mental state attribution task where a target-face with either an ambiguous or an unambiguous emotion is embedded in different social contexts. The social context is determined by the emotions conveyed by other faces in the scene. This task involves mental state attribution to a target-face (either *happy* or *sad*) depending on the social context. Using active inference models, we provide a proof of concept that an agent’s perception of sensory stimuli may be altered by social context. We show with simulations that context congruency and facial expression coherency improve behavioural performance in terms of decision times. Furthermore, we show through simulations that the abnormal viewing strategies employed by patients with schizophrenia may be due to (i) an imbalance between the precisions of local and global features in the scene and (ii) a failure to modulate the sensory precision to contextualise emotions.

## Introduction

We continually make inferences about the state of the world based on the sensory information available to us. However, visual, lexical and semantic obscurities often prevent us from perceiving things for what they are. When this occurs, we must seek out additional information to resolve uncertainty about the true state of the world. Statistical regularities in the world often provide context that we may use to resolve uncertainty about the intrinsically ambiguous observations we make. For example, context resolves uncertainty in the way a sentence resolves uncertainty about a word. A word can have multiple meaning by itself, but the sentence in which it is used imbues it with a definite meaning.

**Figure 1:**
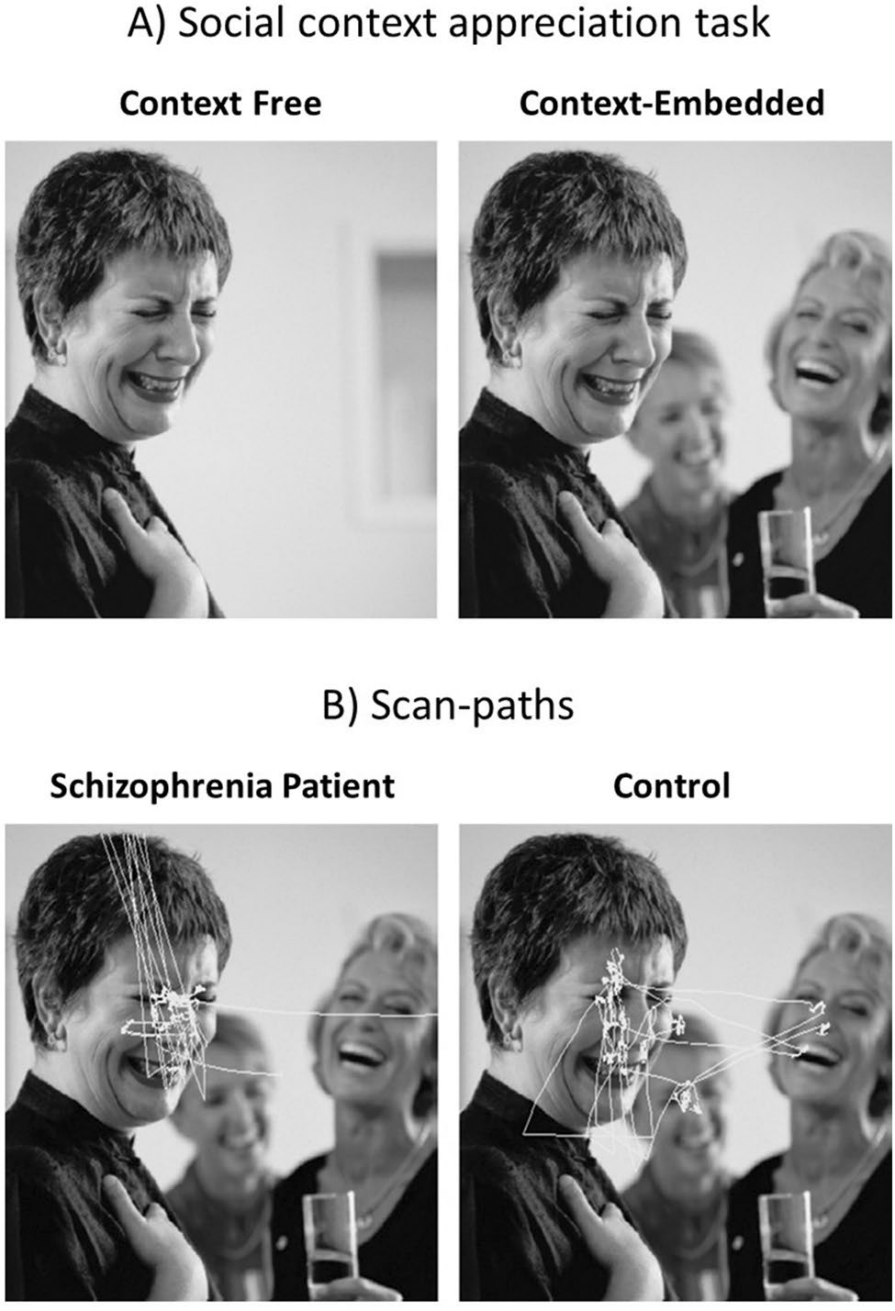
Social context appreciation task. (**A**) This panel shows sample images from the social context appreciation task. A target character is first presented against a background with no contextual information (i.e. context-free image). The same character is then embedded in a social context (i.e. context embedded image). Participants are asked to report their beliefs about what the target-character is feeling or thinking. (**B**) Sample scan-paths of a patient with schizophrenia and healthy participant on a context-embedded image. The panels in this figure are used with permission from Journal of Psychiatry & Neuroscience (http://www.jpn.ca/). These figures are modified from ‘Visual processing of social context during mental state perception in schizophrenia’ J Psychiatry Neurosci 2008;33(1):34–42. Please see^1^ for the original figure.

Contextual information is crucial for mental state attribution. An example of this is the *social context appreciation task*^1^, where a target-character with ambiguous emotion is first presented by itself, and later within a social context (see Fig. 1A). In the context-embedded case, the characters other than the target-character define a social context. As an example, one can infer a target-character that expresses an ambiguous emotion as happy if the faces that define the social context express unambiguously happy emotion. Inferring the emotion of the target-character requires the participants to make an inference about the social context.

In this work, we will use a similar paradigm to the *social context appreciation task* and demonstrate how context can alter perception. This task involves embedding a target-face with either ambiguous or unambiguous emotion in different social contexts (e.g. happy, sad, etc.). By modulating the agent’s confidence in its visual inputs (or sensory precision)^2–4^, we will show that the emotion of a target-character can be perceived differently under different social contexts. In this task, the target-face can be embedded either in an affectively congruent, an incongruent social context or neither. Moreover, the target-face can either have incoherent expressions, resulting in an ambiguous emotion, or it can have coherent expressions that cue an unambiguous emotion. We will show with simulations how the context congruency and coherency of facial features improve visual search performance in terms of decision time and accuracy.

Schizophrenia is a mental disorder that is associated with impairments in several cognitive domains involving perception and attention. These impairments have been studied using paradigms involving visual stimuli as well as other types of stimuli. Visual exploratory behaviour in schizophrenia is often described in terms of shorter scan-paths and fewer fixations^5,6^. Some studies show that these eye-movement components can be improved by intranasally administered oxytocin^7^. In the social context appreciation task, people with schizophrenia were less accurate at attributing a mental state to the target-character and they paid less attention to the contextual cues than healthy controls (see Fig. 1B). Green and colleagues argued that this behaviour represents a tendency to local viewing rather than using a global viewing strategy.

Perceptual differences in schizophrenia might in part be attributable to reduced visual exploration as one might miss out on contextual information which is crucial for correct perception. However, limited visual searches cannot solely explain these differences as perceptual differences have been reported in visual paradigms involving even low-level visual stimuli^8^. For example, patients with schizophrenia demonstrated superior performance, relative to psychiatric controls, at assessing the contrast level of a target object, during a visual illusion task, when the same object is surrounded by a high contrast object. The high contrast object causes the contrast level of the target object to be perceived as lower than when the target object is presented by itself. This study has shown that patients with schizophrenia were less prone to visual illusions as they were not able to utilise contextual information^9^.

In this work, we will simulate visual search patterns to illustrate how the potential belief structures underlying viewing strategies employed in schizophrenia manifest in the *social context appreciation task*. These belief structures induce (i) an inability to utilise contextual information, and (ii) an abnormal allocation of significance to local features relative to global features in the scene.

In this work, we use models that are based on the Bayesian brain hypothesis^10^. Under the Bayesian brain hypothesis, the brain uses a model of the world to infer the most likely hidden causes of sensory information. Beliefs about these hidden causes are updated through Bayesian inference as new sensory information becomes available. The term ‘beliefs’ is used here in the probabilistic ‘Bayesian belief updating’ sense and does not refer to propositional beliefs. Bayesian inference transforms the prior beliefs about the causes (or hypotheses) into posterior beliefs in the light of new sensory data. In particular, we will simulate active inference^11^. Active inference treats the hidden causes of sensory data as hypotheses that can be tested^12,13^ by taking actions and comparing the observed sensations with the sensations expected by the model. Under this framework, perception corresponds to inference on the hidden causes of sensory information, whereas attention corresponds to inferring the precision (inverse variance or negentropy) of sensory signals^14^. In our previous work^15^, we described the computational basis of selective attention as downweighing the precision of context-irrelevant stimuli and maximising the precision of task-relevant stimuli. In the current work, we aim to provide a computational account of contextual perception; using inference about emotional expressions as an illustrative and ecologically important example.

This paper comprises four sections. In the first, we introduce a mental state attribution task, where a target-face with either ambiguous or unambiguous emotion is embedded in different social contexts. We then describe the Markov Decision Process (MDP) model of this task. Subsequently, we introduce a generative model of contextual perception and simulate behavioural responses using the mental state attribution task. We then show that this model reproduces phenomena such as context congruence and feature coherence effects on behavioural measures. We continue with potential computational mechanisms that might account for simulated viewing patterns of schizophrenia and discuss these results in the discussion section. The “Materials and methods” section describes our active inference formalism for MDP and the perception and action cycle in terms of variational message passing. We conclude by expressing the belief structures of the models used in this work in terms of precision matrices.

## Mental state attribution task

We now introduce a visual search task inspired by the *social context appreciation task*^1^, where a target-face is embedded in different social contexts. The target-face can express either a *happy* or *sad* emotion. Furthermore, the target-face can either have coherent facial expressions that convey emotion unambiguously or incoherent facial expressions that convey emotion ambiguously. We describe:

(i) an unambiguously happy face in terms of narrow eyelids and exposed teeth,
(ii) an unambiguously sad face in terms of pulled together eyebrows and covered teeth,
(iii) an ambiguously happy or sad face in terms of pulled together eyebrows and exposed teeth.

**Figure 2:**
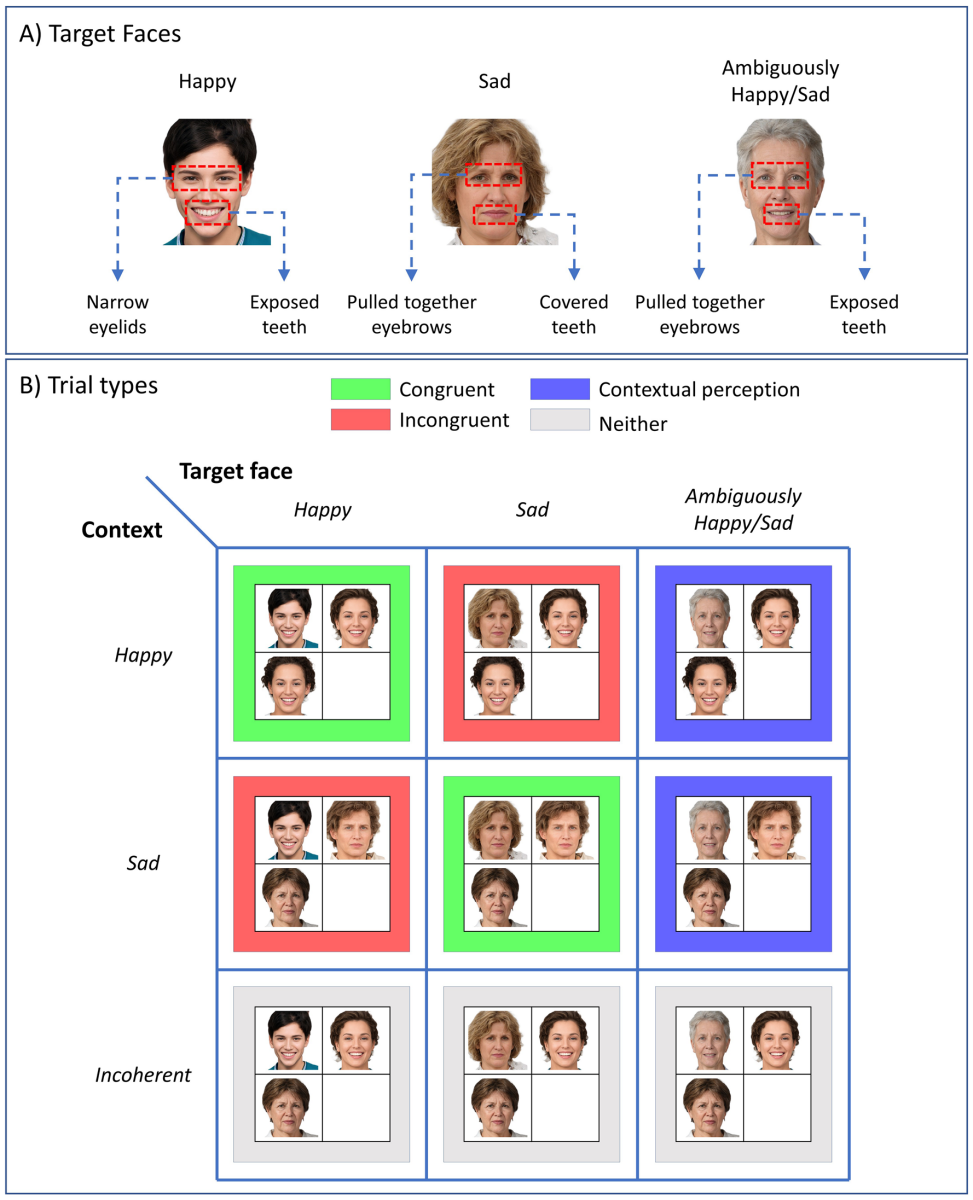
Face and trial types. (**A**)This panel shows the facial expressions of *happy*, *sad* and *ambiguously happy/sad faces*. A *happy* face is expressed by narrow eyelids and exposed teeth. A *sad* face is expressed by pulled together eyebrows and covered teeth. Finally, *ambiguously happy/sad* faces are expressed by pulled together eyebrows and exposed teeth. (**B**) There are four types of trials in the social context attribution task. Trials against the green and red backgrounds are affectively congruent trials (e.g., happy target face in a happy context) and incongruent trials (e.g. happy target face in a sad context), respectively. We expect the context to shape perception when an ambiguously happy/sad target face is embedded in coherent contexts (i.e., faces that define the context are both happy or sad). These are shown against a blue background. Finally, the grey trials show that the target face is embedded in incoherent contexts (i.e., one of the faces that define the context is happy and the other is sad). Photos of faces are used with permission by Generated Photos (https://generated.photos/).

Here, we assumed that the ambiguously happy and sad faces share the same facial expressions. Ambiguously happy/sad faces share one feature with unambiguously happy and sad faces each. From now on we will refer to unambiguously happy and sad faces as happy and sad faces. See Fig. 2A for a visual depiction of each of the faces described above.

Previous work in the literature suggests that different sets of facial muscle movements are involved when different emotions are elicited^16^. For example, a smile with exposed teeth and covered teeth with lowered lip corners are often seen when happy and sad emotions are elicited, respectively. It is also true that similar emotions can manifest slightly different. For example, a happy face can be expressed with a smile with or without exposed teeth. In this work, we used an oversimplified representation of facial expressions of emotions such that narrow eyelids and exposed teeth represent happy emotion, whereas pulled-together eyebrows and covered teeth represent sad emotion. In this simplistic characterisation of happy emotion, narrow eyelids represent a facial feature where the lower eyelids are raised, and exposed teeth represent a smile. Similarly, we use a simplified characterisation of sad emotion, where pulled-together eyebrows give rise to noticeable vertical lines between the eyebrows and covered teeth represent closed lips^16^. These features are merely a subset of facial expressions observed when happy and sad emotions are elicited. In more complex paradigms, one can include different facial expressions and use systems such as the Emotion Facial Action Coding System (EMFACS) to identify facial actions related to emotions^17^.

In this task, there are two other faces in the scene along with the target-face, and they define the social context. These two faces can each express happy and sad emotions.

In our paradigm, there are four types of trials,

(i) Happy/sad target-faces embedded in affectively congruent contexts, see the green panels in Fig. 2B,
(ii) Happy/sad target-faces embedded in affectively incongruent contexts, see the red panels,
(iii) *Ambiguously* happy/sad target-faces embedded in coherent contexts (coherent context: both faces are either happy or sad), see the blue panels.
(iv) Happy/sad and ambiguous target-faces embedded in incoherent contexts (incoherent context: one face expresses happy, whereas the other expresses sad emotions), see the grey panels,

The faces that define the social context will always express happy or sad emotions. However, the target-face can express ambiguous emotions as well. The goal in this task is to identify the emotion of the target-face by sampling the features of the target-face and the social cues determined by the other faces in the scene.

A coherent social context can resolve uncertainty about the emotion of the target face if the target-face consists of expressions that can be attributed to more than one emotion (e.g. happy and sad). An example of this could be seen in the *social context appreciation task*^1^, where a target-face is displayed in a scene with and without a social context. When the faces that define the social context express happy emotions, the target-face can be perceived as happy. However, when the target face is seen by itself it may be perceived as unhappy. In our task, social context can resolve uncertainty about the target-face emotion on blue trials (i.e., emotionally ambiguous target-face embedded in coherent contexts). We expect that the emotion of the target face would be identified more accurately and faster on affectively congruent (green) trials than on affectively incongruent (red) trials. We also expect that the happy and sad target-faces would be identified faster than the emotionally ambiguous target faces (compare the first two columns with the third column in Fig. 2B). Finally, we expect emotionally ambiguous target-faces to be identified faster under coherent contexts (blue trials) than incoherent contexts (rightmost grey trial). We will not compare trials with emotionally ambiguous target faces in terms of accuracy because the inferred emotion of the ambiguous target-face depends on the context in which it is embedded as we will see later.

## Model summary

We used a Markov decision process (MDP) formulation of active inference for the mental state attribution task. MDP models describe the statistical nature of an environment in terms of probability distributions. These models use discrete states to express how categorical outcomes are generated at each unit time. The state transitions are controlled by actions which are chosen from a repertoire of policies. The states at each time step generate outcomes that can be fed back to the model which can, in turn, be used to infer the most likely states of the world.

**Figure 3:**
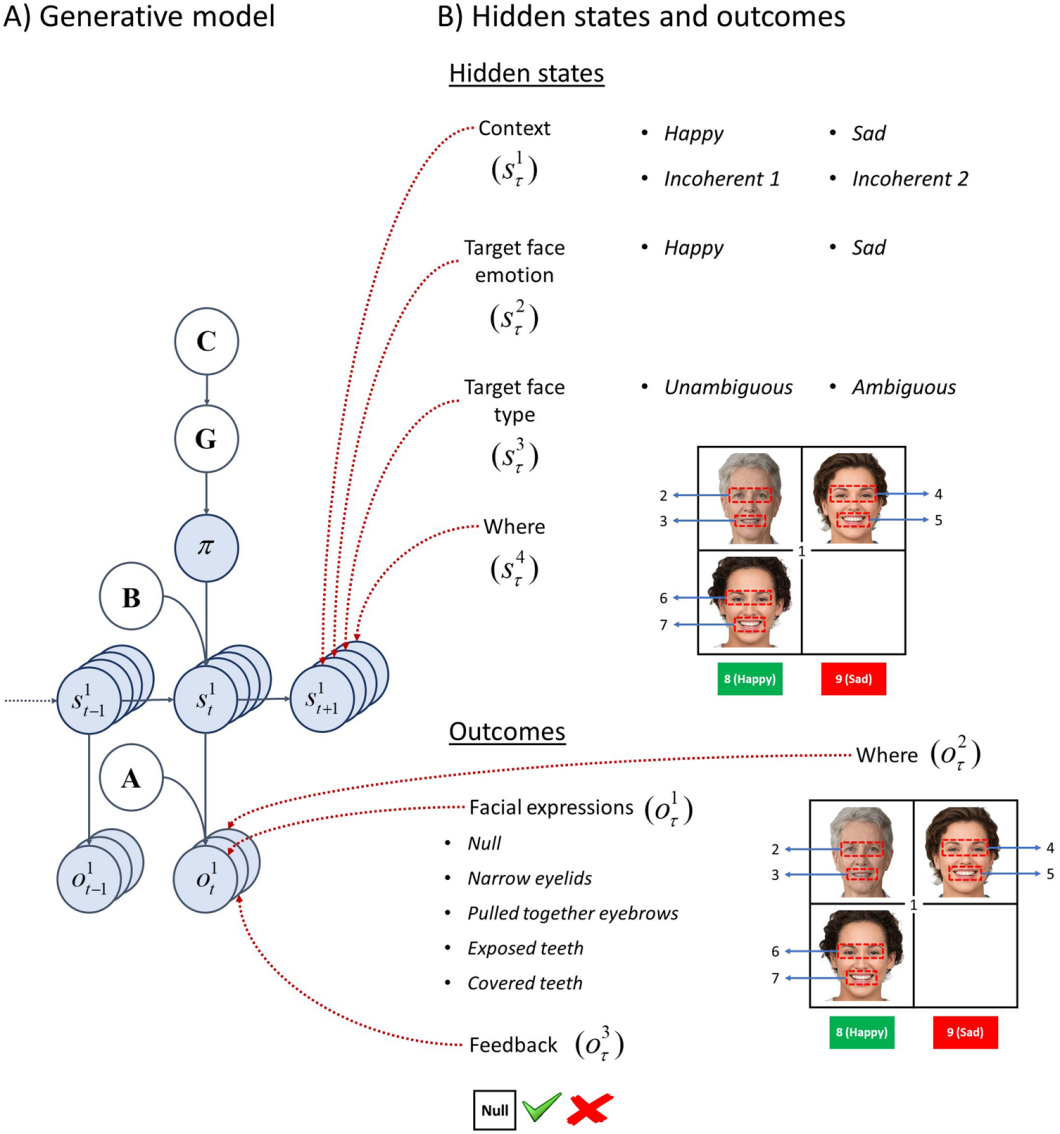
Generative model of the mental state attribution task. (**A**) The left panel shows the dependencies between different units in the generative model. Hidden states ${s}_{t}^{n}$ express latent aspects of the world, where *n* indicates the *n*-th set of hidden states and $t$ indicates time. The state transition matrices $B$ define how likely the states in the next step ${s}_{t+1}^{n}$ are given the states in the current time step ${s}_{t}^{n}$ and the action ${a}_{t}$. A sequence of actions one might pursue in the next time steps is referred to as a policy $\pi$. MDP model considers a repertoire of policies and samples an action from its beliefs about policies at each time step. Active inference describes behaviour in terms of free energy minimisation. A component of free energy is the free energy expected in the future $G$. The policy that minimises expected free energy $G$ is more likely to be pursued. Expected free energy has both extrinsic (pragmatic) and epistemic (information acquiring) components. Extrinsic value is the expected utility under a policy, where the utilities of outcomes are encoded by the prior preference matrix $C$. Epistemic value is the expected information gain about the hidden states. The likelihood matrices $A$ encode how likely an outcome at the current time step ${o}_{t}^{m}$ is given the hidden states in the current time step ${s}_{t}^{n}$. (**B**) This panel shows the hidden state dimensions and outcome modalities considered in the mental state attribution task. There are four hidden state factors, namely *context*, *target face emotion*, *target face type* and *where*. Each factor is comprised of a fixed number of possible states (e.g., *target face emotion* can be happy or sad). In the simulations, the *target face emotion* and *target face type* hidden states were joined (i.e. expressed by a single set of hidden states). We opted to express them by two separate sets of hidden states for ease of clarification. There are three outcome modalities, namely *facial expressions*, *where* and *feedback*. There are several outcomes under each modality (e.g., under the *feedback* modality, the possible outcomes are *null*, *correct* and *incorrect*). Photos of faces are used with permission by Generated Photos (https://generated.photos/).

Active inference explains the behaviour of self-organising systems in terms of free energy minimisation, where free energy is a proxy for surprise. Surprise expresses unexpectedness of an outcome. An outcome can be unexpected (i.e., less likely) for several reasons. One reason might be that the model might have made incorrect inferences about the states of the world and with these false beliefs, the model can take actions that might risk its existence (e.g. approaching a predator that is perceived as friendly). Under the assumption that the living things maximise their chances of survival, a mismatch between the inferred state of the predator (i.e., friendly) and the true state of the predator (i.e., hostile) is likely to yield an unexpected outcome (i.e. a situation that poses a risk to existence). Another reason why surprise may occur is that the model might have false representations of its environment (e.g. not appreciating the law of gravity, one can walk off a cliff without fear). Similarly, having a false representation of the physics of the world, one might face an unexpected outcome. In the MDP models of active inference, the real-world dynamics are described by generative *processes*, whereas the beliefs about these dynamics are described by generative *models*. In this work, we are going to use different generative models (i.e., representations of the statistical structure of the world) to illustrate what types of models might account for the visual search patterns in schizophrenia. See Fig. 3A for the graphical representation of the MDP generative model.

In the mental state attribution task, we considered four sets of hidden states, namely, *context*, *target face emotion*, *target face type* and *where*. Each of these sets of hidden states can be on one level. The *Context* hidden state defines the emotion conveyed by the faces that define the context. There are four levels under the context hidden state, namely happy, sad, and two incoherent contexts. Under the *happy* context, the two faces that form the social context will express happy emotions. Conversely, the *sad* context consists of two faces that express sad emotions. The two incoherent contexts consist of one happy and one sad face each. The only difference between the incoherent contexts is the locations of the happy and sad faces in the top right and lower left cells, which are swapped relative to one another. The *target face emotion* hidden state defines the emotion of the target face which can be either happy or sad. The *target face type* hidden state changes how ambiguously the target face expresses happy and sad emotions. This hidden state has two levels, namely unambiguous and ambiguous. The *where* hidden state has nine levels that correspond to the discrete locations that the model can foveate. The first location is the central fixation. Locations 2 and 3 are associated with the eyes and the mouth of the target face, whereas locations 4 to 7 are associated with the eyes and the mouths of the faces that define the social context. There are two additional locations associated with reporting happy and sad emotions, namely 8 and 9. The model samples one of these locations to report its beliefs about the emotion of the target face.

There are three outcome modalities, namely *facial expressions*, *feedback* and *where*. The first outcome modality, *facial expressions*, signals the facial expressions *narrow eyelids*, *pulled together eyebrows*, *exposed teeth* and *covered teeth* at the locations where facial expressions can be displayed in the scene. There is also a *null* outcome for locations with no facial expressions (e.g. initial fixation). The second outcome modality, *where*, signals the foveated location (i.e. one of nine locations). The third outcome modality, *feedback*, has three outcomes, namely *null*, *correct* and *incorrect*. The correct and incorrect outcomes inform the agent about whether it identified the emotion of the target-face *correctly* or *incorrectly*, respectively. Sampling other locations would return a *null* feedback (i.e., void of feedback). See Fig. 3B for the hidden states and outcome modalities.

**Figure 4:**
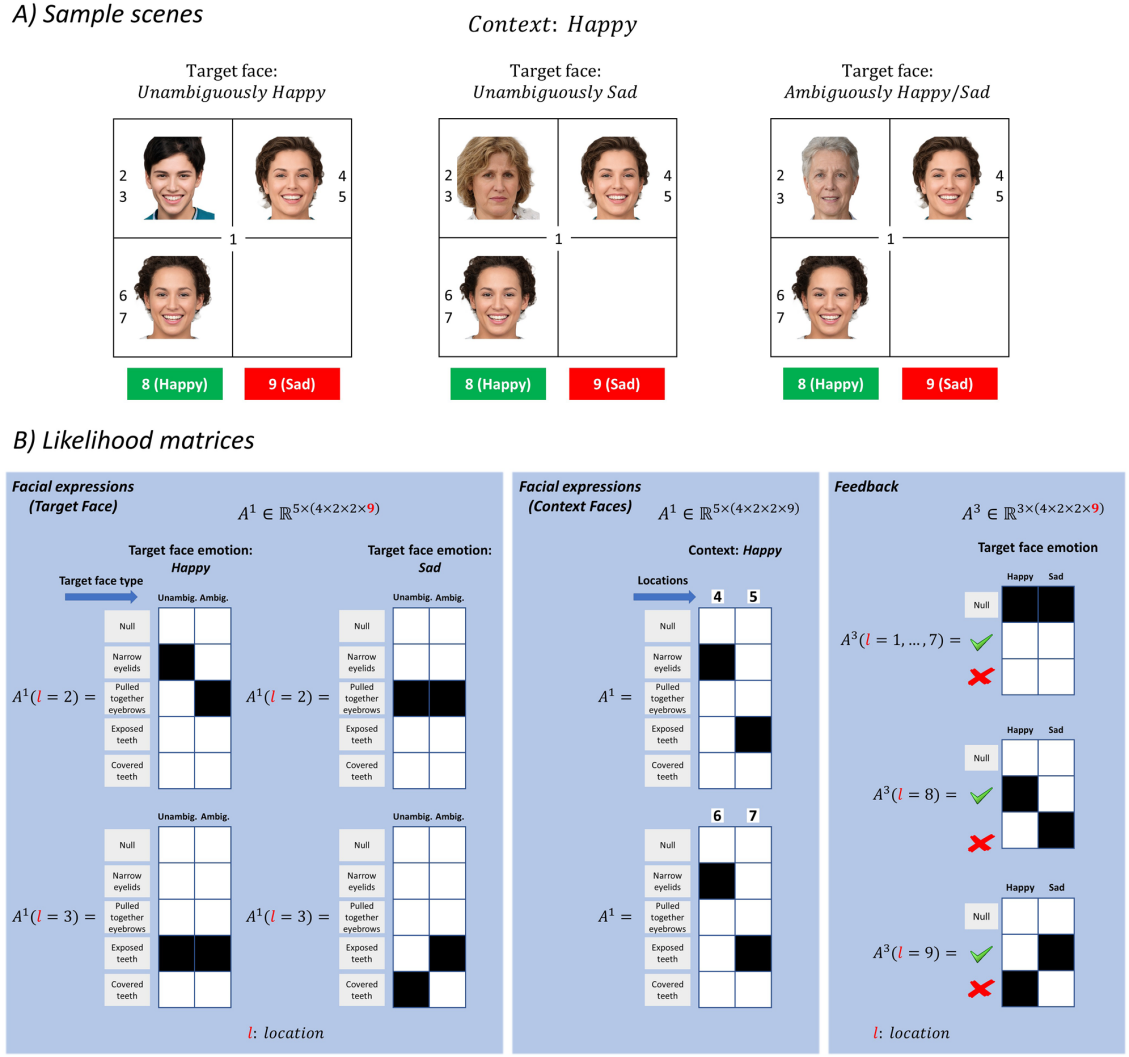
Likelihood matrices. (**A**) This panel shows sample scenes where target-faces (top left quadrants) with happy, sad and ambiguously happy/sad emotions are embedded in happy contexts (defined by the emotions of the faces in the top right and bottom left quadrants). (**B**) These panels show the likelihood matrices ($A$) in the *generative process*. The likelihood matrices in the left panel show the likelihood of the *facial expressions* for unambiguously and ambiguously happy/sad faces. The facial expressions of the target-face depend on the *target face emotion* and *type* hidden states. These expressions are displayed in locations 2 (i.e., eyes) and 3 (i.e. mouth). The panel in the centre shows the likelihood of *facial expressions* for the faces that define the social context. The expressions of these faces depend on the *context*. The panel on the right shows the likelihood of *feedback* outcomes. Sampling locations 1 to 7 returns a null feedback (void of feedback), whereas sampling locations 8 and 9 returns *correct* and *incorrect* feedback, respectively, when the target-face is happy. Photos of faces are used with permission by Generated Photos (https://generated.photos/).

We will now describe the likelihood of outcomes in the generative process using the likelihood matrices. Each entry in the likelihood matrix corresponds to the likelihood of an outcome given the hidden states $P\left({o}_{t}|{s}_{t}\right)$. In the MDP model, *context* hidden state maps onto the *facial expressions* (e.g. narrow eyelid, exposed teeth, etc.) that can be seen in locations 4 to 7 (see the upper panels of Fig. 4A). The *target face emotion* and *target face type* hidden states determine the facial expressions of the target-face. For example, if the *target face emotion* is happy and *target face type* is unambiguous, one would see narrow eyelids and exposed teeth at locations 2 and 3, respectively (see Fig. 4A left panel). If the *target face emotion* is sad and the *target face type* is unambiguous, one would see pulled together eyebrows and covered teeth at locations 2 and 3, respectively (see Fig. 4A middle panel). However, if the *target face type* is ambiguous, regardless of the *target face emotion* (i.e., either happy or sad), one would see pulled together eyebrows and exposed teeth at the same locations (see Fig. 4A right panel). The co-occurrence of the facial expressions *pulled together eyebrows* and *exposed teeth* define ambiguously happy and sad faces. This is because pulled together eyebrows are associated more with the sad emotion than happy and exposed teeth are associated more with the happy emotion than sad. Clearly, this is a simplification of the complex relationship between affective states and their skeletomotor manifestations^18^. This simplification is deliberate such that we have everything we need for the points we seek to make without overcomplicating these issues. The left panel of Fig. 4B shows the likelihood of *facial expressions* for target-faces. These matrices are expressed for the locations 2 and 3 (i.e. locations where the target face can appear) and the columns of the matrix are the different levels of *target face emotion* (i.e., happy or sad) and *type* (i.e., unambiguous or ambiguous). The middle panel shows the likelihood of *facial expressions* but this time for the faces that define the social context. These matrices are expressed for the locations 4 to 7 (i.e., the locations where the faces that define the social context can appear). *Where* outcomes depend only on *where* hidden states and there is an identity mapping from *where* states to outcomes (not shown for simplicity). *Feedback* depends on the *target face emotion* and *where* (sampled location) hidden states. If the *target face emotion* is happy, visiting location 8 (i.e., the location associated with happy emotion) would return a correct feedback regardless of whether the *target face type* is unambiguous or ambiguous. However, visiting location 9 (i.e., the location associated with the sad emotion) would return an incorrect feedback for the same setup. Visiting any other location in the scene would return a *null* feedback (see Fig. 4B right panel). As a summary, the sizes of the likelihood matrices are ${\mathbb{R}}^{5\times (4\times 2\times 2\times 9)}$ under *facial expressions*, ${\mathbb{R}}^{9\times (4\times 2\times 2\times 9)}$ under *where* and ${\mathbb{R}}^{3\times (4\times 2\times 2\times 9)}$ under *feedback* outcome modalities. This means that there are $4\times 2\times 2\times 9=144$ state combinations giving rise to outcomes under three separate modalities.

Prior preference matrices express how much the agent prefers one outcome over another. In this task, we only defined preferences over the correct (utility of $0.5$ relative log probability of 0.5 nats) and incorrect (utility of − $10$) outcomes in the *feedback* modality. These utilities encourage the agent to report its beliefs about the emotion of the target-face once it has accumulated enough evidence.

The state transition matrices for the *context*, *target face emotion* and *target face type* hidden states are all identity matrices. With these transition matrices, the social context and the facial expressions of the target-face do not change in the course of a trial. The only state that the agent can change and has control over is the location it can sample. This is enabled by the policy dependent *where* matrices (which are zero everywhere except for of a row of ones for the corresponding action). As an example, the agent would look at the eyes of the target-face if the sampled policy is ‘2’ regardless of where it sampled in the previous time step. A policy is a sequence of actions that specifies which action will be chosen in each future time step. In our simulations, we chose the policy depth as one, meaning that there was only one (unique) action under each policy. The posterior beliefs about the policies depend on expected free energy which has two main components. The first component, epistemic value, expresses how much information is expected to be acquired about the hidden states under a given policy. Epistemic value expresses how the beliefs about the hidden states would change when expected outcomes (under a policy) are considered. As an example, let us assume that we want to find out whether it rains outside. We have two options (or policies), namely entering a room with or without a window. Entering a windowless room would not afford any information about whether it is raining outside as the visual observations we expect to make there, are not relevant to whether it rains outside. The second component, extrinsic value, expresses how likely a given policy is to produce the outcomes that the agent prefers. An example is, if we want to have a snack, we will go to the kitchen. These two components express how good a policy is. Applying a softmax function to (negative) expected free energy yields the posterior beliefs over the policies. In other words, policies are probabilistically defined by the expected Free Energy under each scenario. An action is then sampled from the posterior beliefs over the policies. Actions can be chosen in one of two ways. The first is sampling an action from the maximum a posteriori (MAP) estimate of policies, which corresponds to choosing an action from the policy with the greatest posterior probability. The second is sampling an action stochastically. The latter is useful in situations where we want to preclude ceiling effects arising from high posterior probability associated with a policy. In this work, we used the MAP estimate of policies to choose actions unless otherwise is stated in figure legends.

In the next section, we will introduce precision parameters associated with the mapping from hidden states to outcomes in the likelihood matrix. These parameters determine how informative outcomes are about the hidden states that generate them. Precision parameters correspond to the inverse temperature parameters of softmax functions^19^. The equation below shows how these precision parameters are applied to likelihood matrices,

1 $$P(o_{\tau }^{m} = j|s_{\tau }^{n} = i,\zeta_{i} ) = \frac{{\left( {A_{ji}^{m} } \right)^{{\zeta_{i} }} }}{{\sum\limits_{k} {\left( {A_{ki}^{m} } \right)^{{\zeta_{i} }} } }}$$

Here, ${o}_{\tau }^{m}=j$ and ${s}_{\tau }^{n}=i$ correspond to the *j*-th outcome under the *m*-th outcome modality and *i*-th hidden state under the *n*-th hidden state factor, respectively. The precision term ${\zeta }_{i}$ modulates the likelihood mapping from the *i*-th hidden state under the *n*-th hidden state factor to the outcomes under the *m*-th outcome modality. The lowest possible precision parameter (e.g., ${\zeta }_{i}\approx 0$) would change the likelihood mapping to outcomes such that observing an outcome would not acquire any information about that hidden state. A high precision parameter, on the other hand, (e.g., ${\zeta }_{i}\gg 0$) would yield a deterministic mapping from a hidden state to an outcome and allow the model to make precise inference about the hidden states. In paradigms involving multiple outcome modalities and hidden state factors, it is useful to express the mapping from hidden states to outcome modalities in terms of precision matrices. The role of the precision matrix can be demonstrated with the toy ‘rain’ example we used when describing epistemic value;2

Here, the states correspond to rooms with and without a window. The precision vector above expresses beliefs such that in a windowless room, there is no way of finding out whether it rains. However, entering a room with a window would give precise information about whether it rains. These beliefs are expressed with the precision terms ${\zeta }_{window}=\infty$ and ${\zeta }_{windowless}=0$, which are shown above as entries of a vector mapping from hidden states to the outcome modality *Rain*. It is important to note that these precision parameters operate on the generative model likelihood matrices in our paradigm. We will use precision parameters to show how they can enable contextual perception.

## Simulations

In the social context attribution task, social cues resolve uncertainty about the target character’s emotional state. An example of this can be seen in Fig. 1A, where the target-character is inferred as happy when the social context conveys happy emotion. In this section, we will first simulate visual search patterns with a model that can attribute a mental state to the target-face based on the context. We will then show with simulations that this model reproduces some of the well-known phenomena such as context congruence and feature coherence effect on decision times and accuracy rates. Finally, we will use different models to describe the computational mechanisms underlying visual search patterns in schizophrenia.

**Figure 5:**
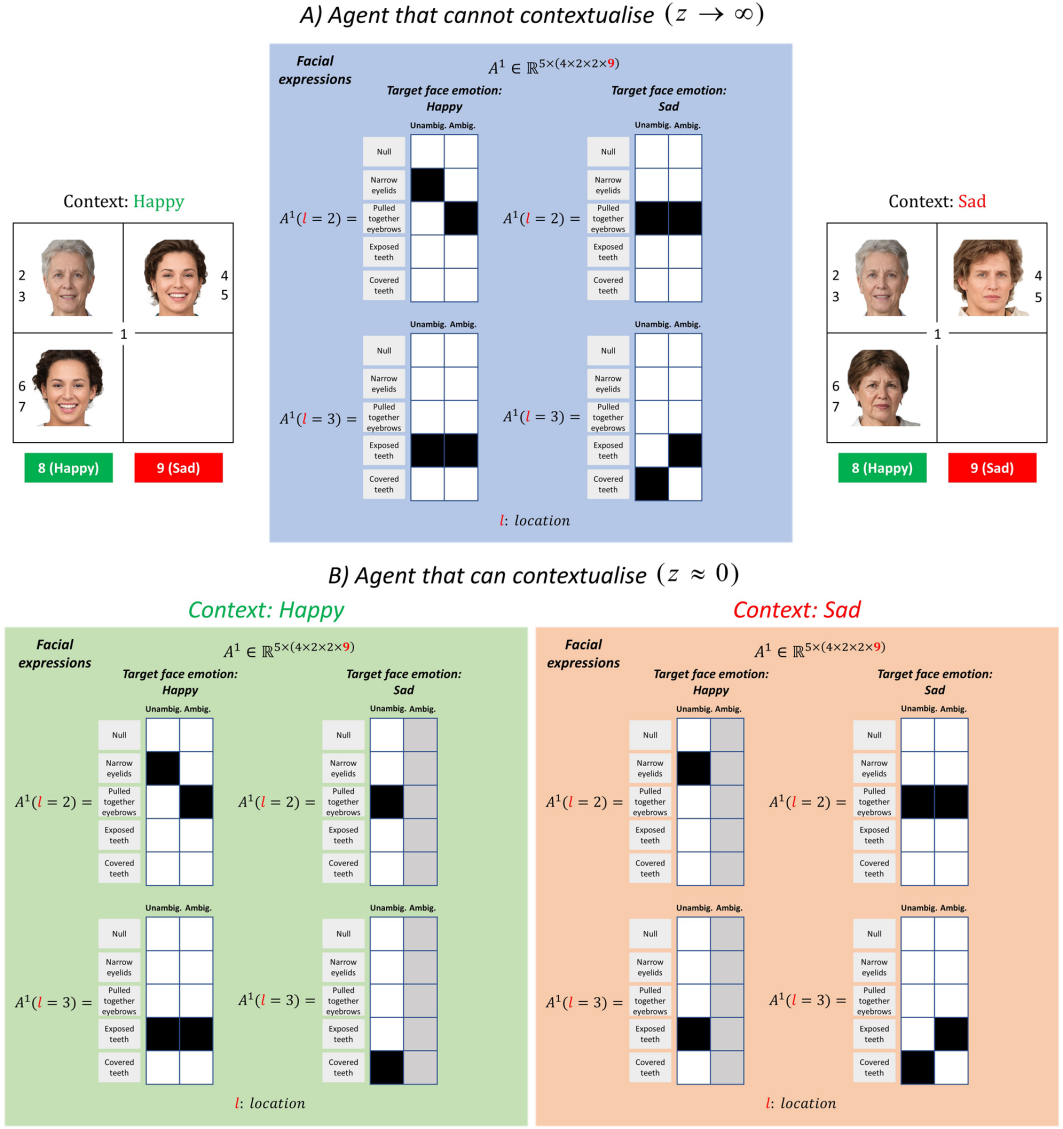
Contextual perception and precision. (**A**) This panel shows the *generative model* likelihood matrices for an agent that cannot contextualise target-face emotion. The likelihood matrices for the facial expressions shown here are identical under all contexts (e.g., happy, sad, etc.) when the precision parameter is high ($z\to \infty$). Notice that these matrices are identical to the *generative process* likelihood matrices for the facial expressions (see Fig. 4B left panel). (**B**) These panels show the same likelihood matrices for an agent that can contextualise the target-face emotion ($z=0$). The agent cannot associate the facial expressions of ambiguously sad faces with the sad emotion when the context is happy (see the matrices on the right under happy context). Similarly, the agent cannot associate the facial expressions of ambiguously happy faces with the happy emotion when the context is sad (see the matrices on the left under sad context). Photos of faces are used with permission by Generated Photos (https://generated.photos/).

In our version of the task, we assumed that the emotionally ambiguous target-faces should be inferred as happy and sad when the context conveys happy and sad emotions, respectively. In other words, the agent should expect the emotion of the target-face to be consistent with the context. Figure 5A shows a set of likelihood matrices for the facial expressions of the target-face given hidden states. These matrices represent the prior beliefs of an agent that cannot utilise contextual information to attribute a mental state to target-faces. These matrices are identical under all contexts (i.e., happy, sad, incoherent contexts). This means that when the agent sees pulled together eyebrows and exposed teeth (i.e. ambiguous emotion), the agent is equally likely to attribute these features to happy and sad emotions. However, the likelihood matrices of the agent that can utilise contextual information are context-specific (see Fig. 5B). This agent can attribute the very same features uniquely to happy and sad emotions under happy and sad contexts, respectively. From a computational perspective, contextual perception can be modelled by adaptively assigning a relatively lower precision ($z\approx 0$) to the facial expressions of ambiguously sad target-faces under the happy context than facial expressions of the ambiguously happy target-face in the generative model (compare the uniform distribution, represented by the grey column, with the precise distribution under *ambiguous* hidden states for the happy context in Fig. 5B). A similar mapping can be seen under the sad context (see “Materials and methods” for more details).

**Figure 6:**
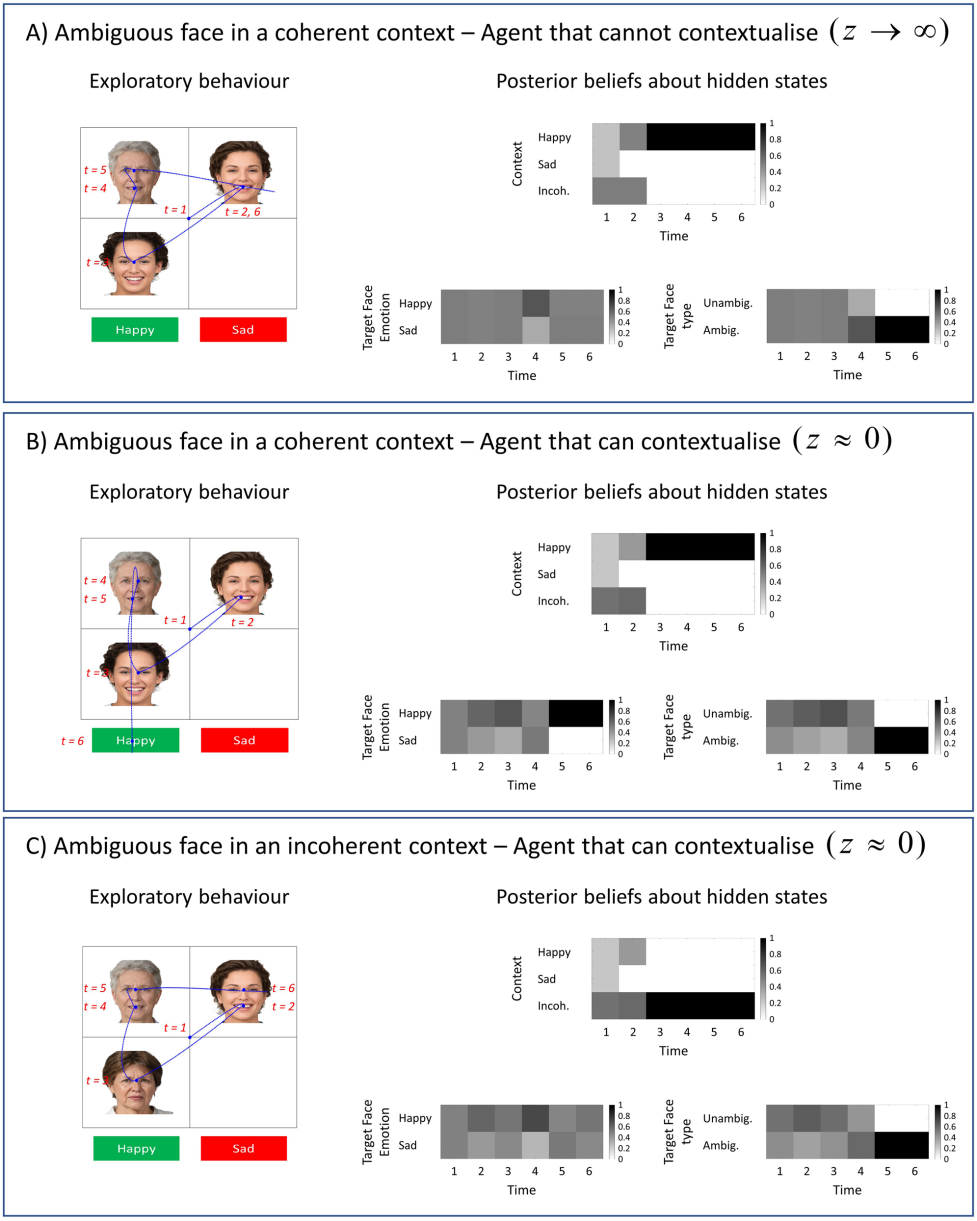
Agents that cannot and that can use contextual information. The left panels show the exploratory behaviours of agents that cannot and that can utilise contextual information. The blue curves show the locations and the order at which these locations are sampled. The correct emotion of the target-face is happy (location 8). The panels on the right show the agent’s posterior beliefs about the *context*, *target face emotion* and *target face type* at each time step. The beliefs about the two incoherent contexts are summed and reported as one incoherent context under the posterior beliefs about context. This is why the incoherent context appears twice as likely as happy and sad contexts at the initial time step. (**A**) This panel shows the exploratory behaviour of an agent that cannot use social information to contextualise target-face (i.e. $z\to \infty$). Here an ambiguously happy target-face is embedded in a happy context. (**B**) This panel shows the same as panel A but this time for an agent that can use social information to contextualise target-face (i.e. $z\approx 0$). C) This panel shows the responses of an agent that can contextualise the target-face emotion (i.e. $z\approx 0$). This time an ambiguously happy target-face is embedded in an incoherent context (i.e. one face is happy the other is sad). We used the MAP estimate of policies for the simulations in this figure. Photos of faces are used with permission by Generated Photos (https://generated.photos/).

Now, we will compare an agent that can and cannot utilise contextual information to attribute an emotion to the target face. The left panels of Fig. 6A,B show the visual scan-path of these agents. Both agents explore a scene where an ambiguously happy target-face is embedded in a happy context (i.e. blue trial in the first row in Fig. 2B). The right panels show the agent’s beliefs about the *context*, *target face emotion* and *target face type* hidden states. It is worth remembering that, in our paradigm, the faces that define the social context do not express ambiguous emotions. This means that seeing only one facial expression (either the expression in the eyes or the mouth) is enough to determine the emotion (either happy or sad) of the faces that form the social context. In all simulations, the agent starts exploring the scene from the central fixation ($t=1$).

Figure 6A shows the simulated responses of an agent that cannot utilise contextual information. At the second time step, the agent visits the face at the top-right cell and finds exposed teeth. After seeing exposed teeth, the agent knows that it observed a *happy* face, and it rules out the possibility of a *sad* context ($t=2$). In the next time step, the agent attends to the face at the bottom-left and finds narrow eyelids—a facial expression that is also associated with the *happy* face. The agent has seen two happy faces so far, and it believes that the social context is *happy* ($t=3$). Then, the agent visits the target-face (top left), where it finds exposed teeth and pulled together eyebrows. Now the agent knows that the target-face expresses an ambiguously *happy*/*sad* emotion ($t=5$). This agent is unable to attribute the *happy* emotion inferred from contextual cues to the target face. Finally, the agent samples one of the locations in the scene randomly, failing to identify the emotion of the target-face ($t=6$).

The agent that can utilise contextual information (see Fig. 6B), similarly explores the faces at the top-right and bottom-left cells and infers the social context as *happy* ($t=3$). The agent also starts to believe that the target-face is *happy* because it believes the social context is *happy*. The agent then visits the target-face and infers it as emotionally ambiguous. This agent can attribute the happy emotion inferred from the context to the target face ($t=5$). Consequently, the agent reports its beliefs about the emotion of the target-face as *happy* and receives a correct feedback ($t=6$). It is worth mentioning that the correct or incorrect feedbacks depend on the *target face emotion* (i.e., happy or sad) and *where* the agent samples in the scene to report its beliefs about the emotion of the target face (i.e., locations 8 and 9 for happy and sad, respectively, akin to ‘pressing the correct button’). The hidden state *target face type* (i.e., ambiguous or unambiguous) is inconsequential for the feedback modality. In our model, we assumed that ambiguously happy/sad faces are identical in terms of features. This means that an agent can contextualise the emotion of these faces as happy under a happy context and sad under a sad context. Importantly, an agent can contextualise an ambiguously sad face *incorrectly* as happy under a happy context. It is plausible that one might incorrectly attribute a happy feeling to someone just because of the context under which they interacted. It is also worth acknowledging that ‘correctness’ depends upon the specific choices we have made when designing the task, and the simplifications we have made. In a more ethologically valid social setting, it might be that the correctness of an inference is judged against other aspects of the other person, including their verbal report about how they feel.

In Fig. 6A,B, we opted to show those simulations where the agent samples the same visual cues in a similar order to show the difference between agents that can and cannot contextualise, in terms of their behaviour. In these simulations, visual search behaviour is initially dominated by epistemic drives to resolve uncertainty about the hidden states (i.e., *context*, *target-face emotion*, *target-face type*). Comparing Fig. 6A,B, we see that even though these two agents sample the same visual cues in the first five timesteps, they infer the target-face emotion differently. The difference between these two agents is expressed in terms of their precision parameters (i.e., *z*). These simulations show that the precision parameter has direct and indirect consequences on agents’ beliefs and behaviour. With a low precision parameter (i.e., $z\approx 0$), the agent that can utilise contextual information infers the *target-face emotion* as happy at $t=5$ (see Fig. 6B). Comparing this agent with the one that cannot utilise contextual information (see Fig. 6A), we see that the parameter *z* is directly responsible for the inferred *target-face emotion* in Fig. 6B. Having inferred the *target-face emotion* as happy (Fig. 6B), extrinsic value (i.e., goal-directed behaviour) takes over this agent’s visual search behaviour, and the agent reports the *target-face emotion* as happy at $t=6$. This shows that the parameter *z* is indirectly responsible for the reported *target-face emotion*.

In the next simulations, we used the agent that can utilise contextual information. Figure 6C shows the simulated responses when an emotionally ambiguous target-face is embedded in an incoherent context (i.e., one face is happy, the other is sad). The agent visits the face at the top-right and finds exposed teeth—a facial expression that is associated with a happy face. Then, the agent visits the face at the bottom-left and finds pulled together eyebrows—a facial expression that is associated with a sad face. The agent has observed one happy and one sad face so far and inferred the context as incoherent. In the next time steps, the agent visits the face at the top left and finds a target-face with ambiguous emotion. Although this agent can utilise contextual information under coherent contexts (i.e., two faces expressing the same emotion), it is unable to contextualise the target-face emotion because the social context is incoherent (i.e., provides inconsistent information). Comparing Fig. 6B,C shows that the agent is faster at attributing an emotion to emotionally ambiguous target-faces when they are embedded in coherent contexts (blue trials in Fig. 2B) than in incoherent contexts (rightmost grey trial in Fig. 2B).

**Figure 7:**
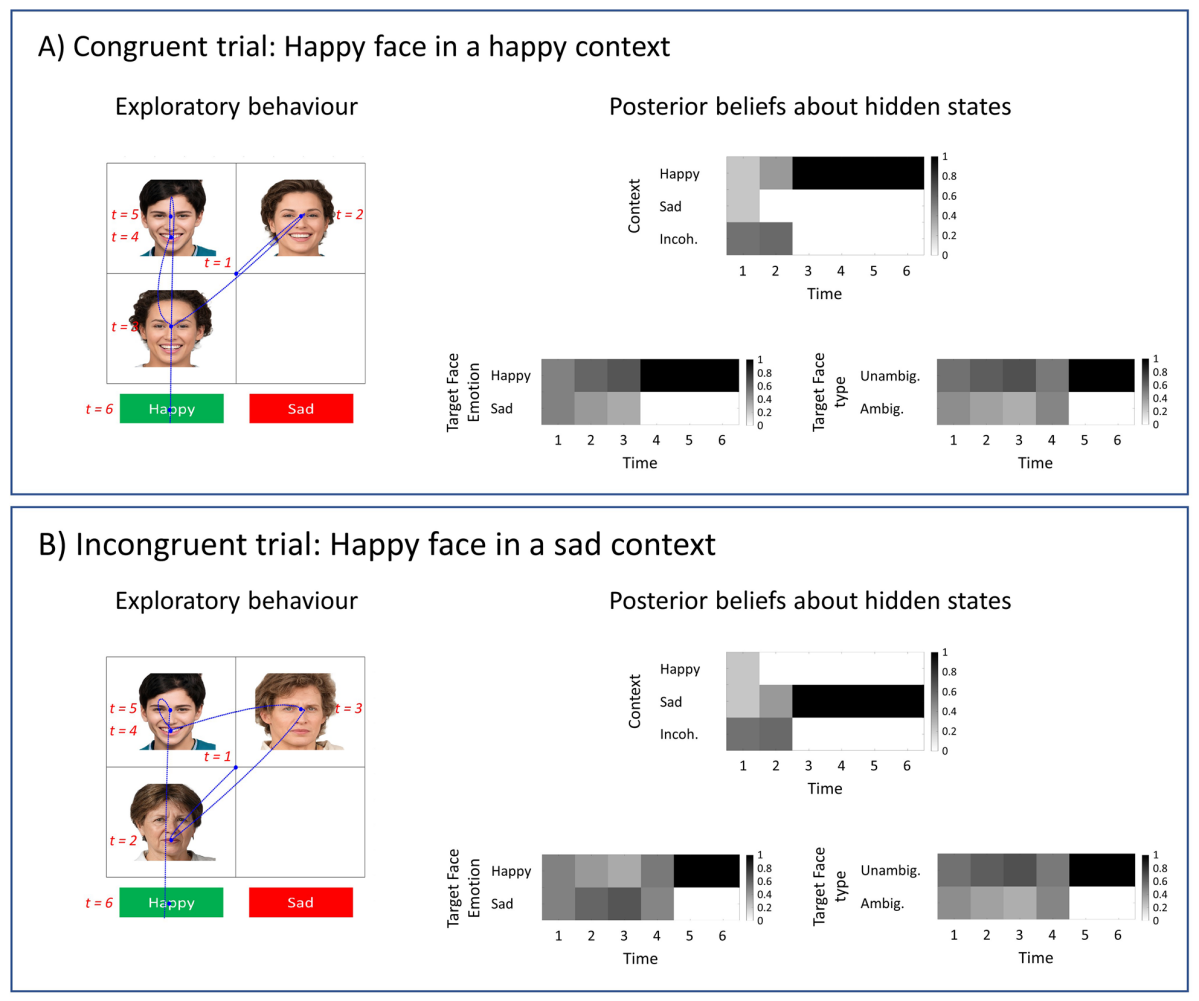
Congruent versus incongruent contexts. The simulated responses in this figure is obtained with an agent that can utilise contextual information. The target-face expresses an unambiguously happy emotion in all panels. (**A**) In this panel, the context is happy (i.e., congruent with the emotion of the target-face. (**B**) The context in this panel is sad (i.e., incongruent with the emotion of the target-face). We see that the agent is able to identify the emotion of the target-face as happy in all panels. Under a congruent context (panel **A**), the agent’s beliefs about the target-face emotion converge to happy emotion faster than in incongruent (panel **B**) context. We used the MAP estimate of policies for the simulations in this figure. Photos of faces are used with permission by Generated Photos (https://generated.photos/).

The same agent identifies the emotion of the target-face as happy when a happy target-face is embedded in happy (congruent) and sad (incongruent) contexts (see Fig. 7A,B, respectively). Notice that the agent believes that a happy face is increasingly more likely under a happy context (see Fig. 7A) and increasingly less likely in a sad context (see Fig. 7B) as it attends to the social cues. These simulations show how different contexts affect the agent’s beliefs about the target-face in the course of a trial. In this section, we opted to show the simulated responses when the target-face expresses a happy emotion; however, one can produce similar results when the target-face is sad as well.

In these simulations, we used the MAP estimate of policies to determine the next location to sample. The order of the sampled locations can vary between simulations as multiple policies can afford the same amount of information about the hidden states. This means that there could be more than one policy with the greatest posterior probability, and in such cases, we randomly choose between these policies. This is why the scan-paths in some simulations are different (see Fig. 7A,B).

### Effect of context congruence and facial feature coherence

**Figure 8:**
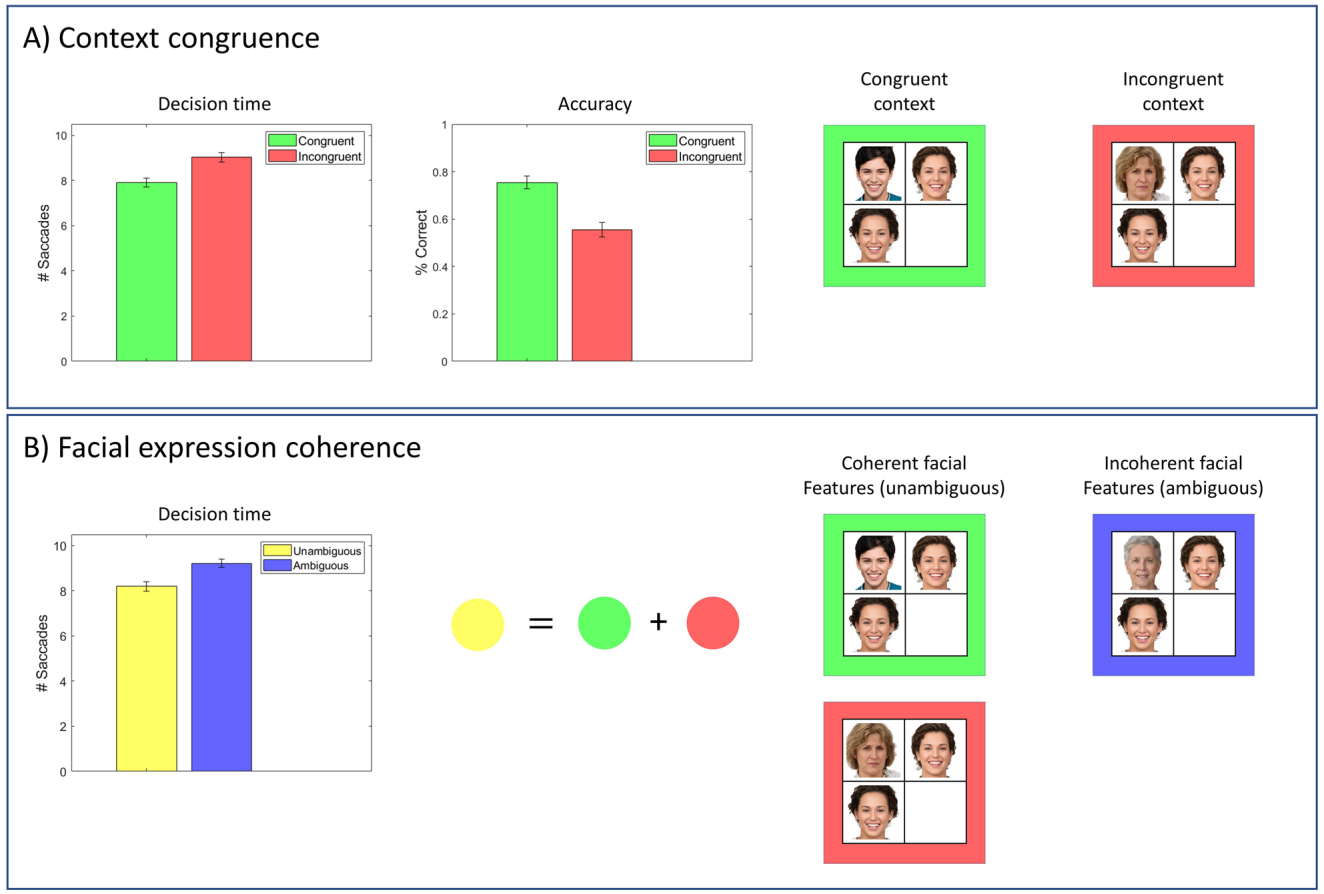
Hypotheses about decision times. (**A**) This panel compares the agent’s behavioural performance between congruent and incongruent trials in terms of decision times and accuracy. (**B**) This panel compares the agent’s performance on trials with ambiguous and unambiguous target-faces. For these simulations, we stochastically sampled an action from the posterior distribution over the policies, rather than choosing the maximum a posteriori (MAP) estimate as shown in Eq. (22) (see “Materials and methods”). This precludes ceiling effects and allows us to see the differences between different conditions. Photos of faces are used with permission by Generated Photos (https://generated.photos/).

Next, we examined the agent who can utilise contextual information to test whether different stimuli elicit different decision times and accuracies. We expect that the agent would attribute emotions to the target-face more accurately and faster when the target-face is embedded in affectively congruent contexts. As expected, context congruency improved the agent’s behavioural performance in terms of decision times and accuracy rates (see Fig. 8A). Decision time is the number of locations that the agent visits before reporting its beliefs about the emotion of the target-face. Accuracy rate is the percentage correct identification of target-face emotion.

Similarly, we expect the agent to perform better when the target-face has coherent facial expressions. Ambiguously happy/sad target-face has incoherent features as it shares one feature with happy and sad faces each (i.e., exposed teeth and pulled together eyebrows). However, happy and sad target-faces have coherent facial features as they do not share any features. Facial feature coherence improved the agent’s performance in terms of decision times (see Fig. 8B). We did not compare these two cases in terms of accuracy as the inferred emotion of the ambiguous target-face depends on the context in which it is embedded.

### Simulating schizophrenia

**Figure 9:**
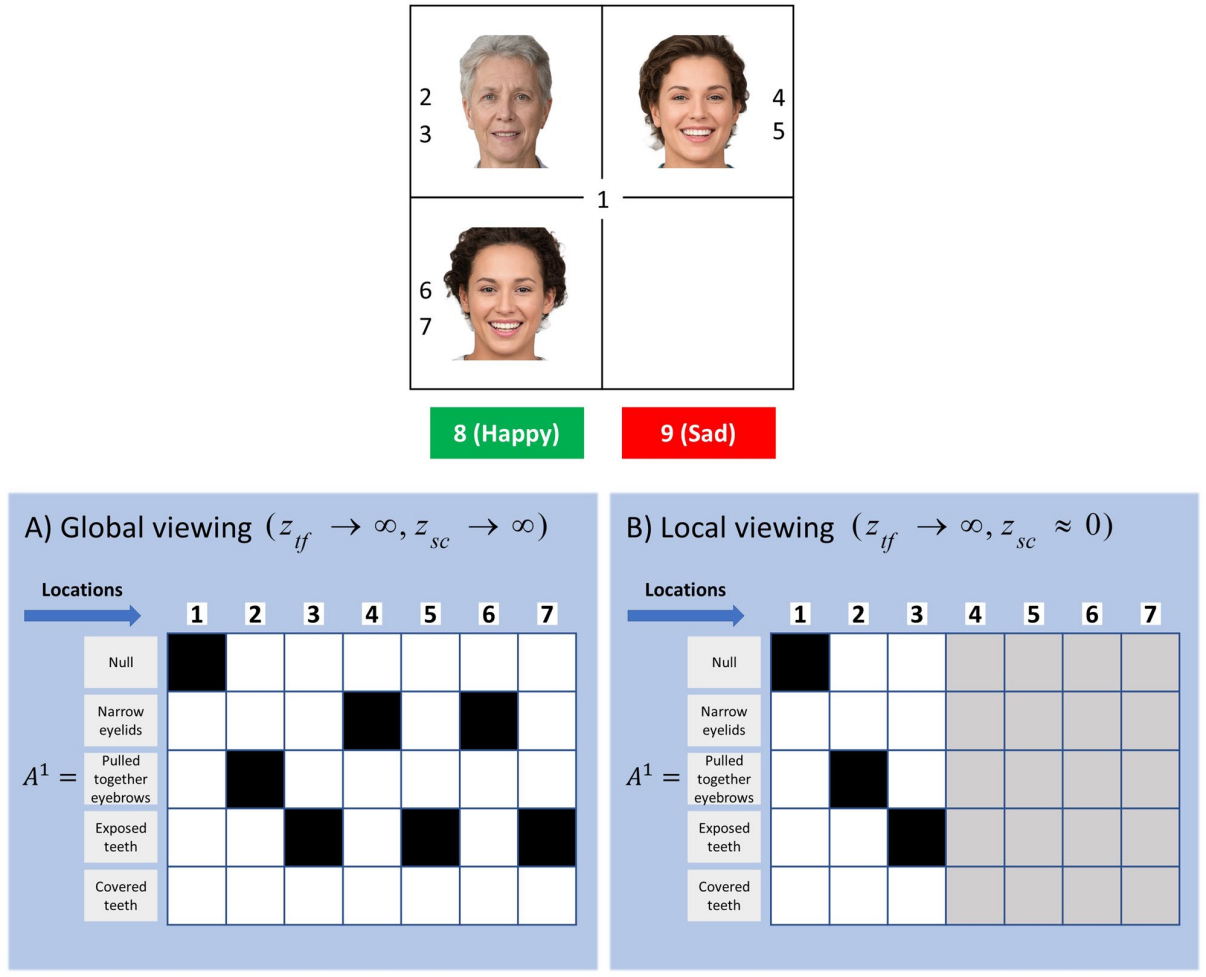
Belief structures underlying global and local viewing strategies. The likelihood matrices in this figure are expressed with respect to the locations in the scene. (**A**) In this panel, the precisions associated with the contextual cues and the target-face are both set high (${z}_{sc}\to \infty , {z}_{tf}\to \infty$). With these precisions the agent values the contextual cues and target-face equally and employs a global viewing strategy. (**B**) In this panel, the precisions associated with the contextual cues is set low relative to the precision associated with the target face (${z}_{sc}\approx 0, {z}_{tf}\to \infty$). The imbalance in relative precisions induces a local viewing strategy. Photos of faces are used with permission by Generated Photos (https://generated.photos/).

**Figure 10:**
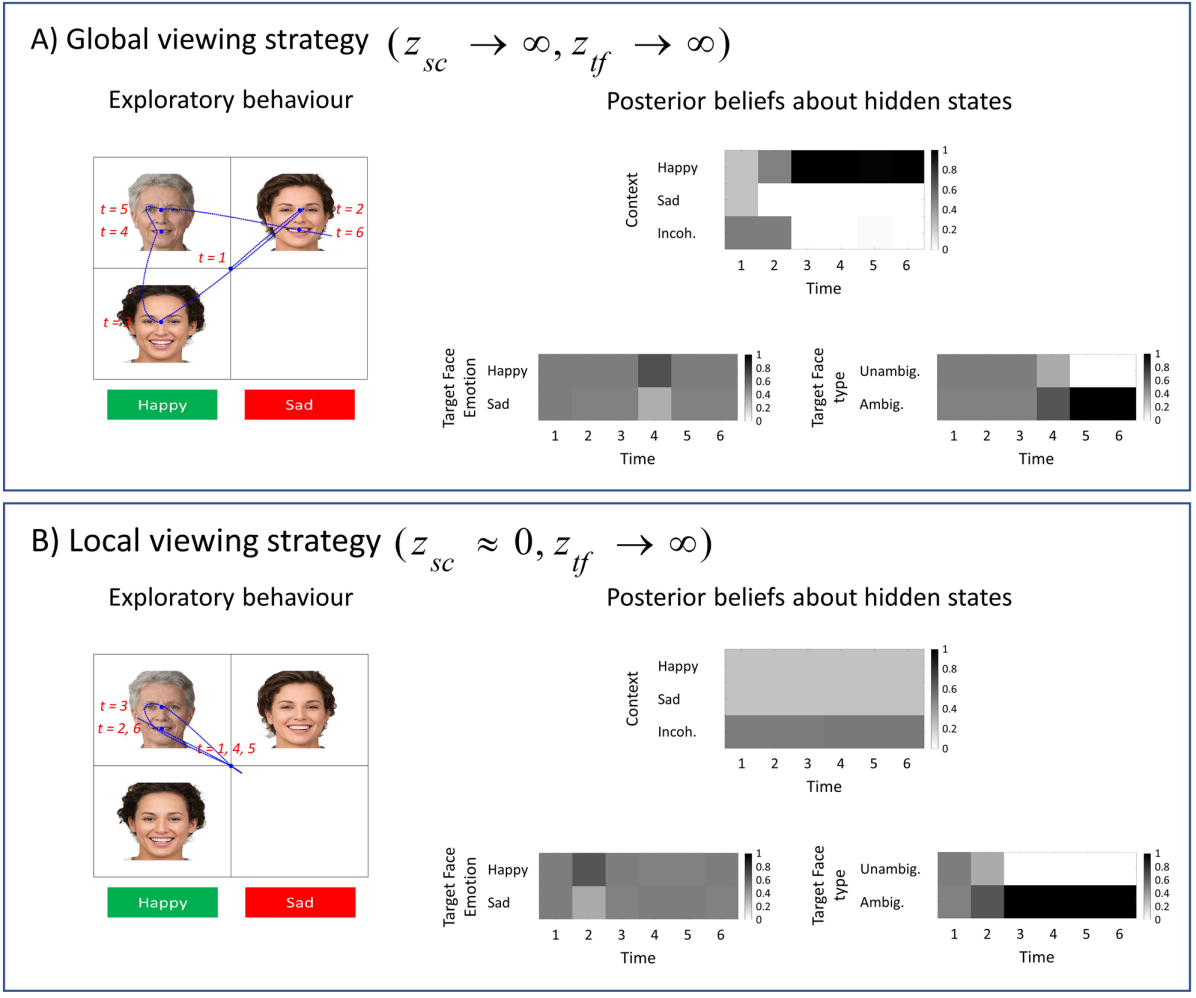
Simulating schizophrenia. The visual search patterns in this figure are obtained with agents that are unable to contextualise the target-face emotion ($z\to \infty$). For both of the simulations, an ambiguously happy/sad target-face is embedded in a happy social context. (**A**) This agent attends to the social cues and the target-face (i.e., ${z}_{sc}\to \infty$ and ${z}_{tf}\to \infty$), however it is still unable to identify the emotion of the target-face as it is unable to utilise contextual cues ($z\to \infty$). (**B**) This agent does not attend to the social cues due to an imbalance between precisions associated with the target-face and social cues (i.e., ${z}_{sc}\approx 0$ and ${z}_{tf}\to \infty$). We used the MAP estimate of policies for the simulations in this figure. Photos of faces are used with permission by Generated Photos (https://generated.photos/).

In the *social context appreciation task*, patients with schizophrenia were less accurate at attributing a mental state to the target character and their visual search behaviour tended to be more localised around the target-character than healthy controls (see Fig. 1B). This behaviour might represent a tendency to local viewing rather than using a global viewing strategy as discussed in Green and colleagues’ work^1^. From a computational perspective, this can be modelled as a decrease in the precision of contextual cues. In other words, this is a belief that data from the object of interest is overwhelmingly more informative than data from other sources. There is a sense in which this is circular, as the most informative data are those that attract interest. From this perspective, manipulating the precision of contextual cues can be thought of as limiting the focus of attention or the range of interest. In our paradigm, this corresponds to an imbalance between the precision of the expressions of the target-face ${z}_{tf}$ and social cues ${z}_{sc}$. Figure 9 shows the likelihood matrices for the facial expressions under two models as a function of locations. Locations 2 and 3 are associated with the facial features of the target-face, whereas locations 4 to 7 are associated with the facial features of the faces that define the social context (see the upper panel in Fig. 9). Figure 9A shows the likelihood matrix for a model that believes that the contextual cues are informative. This model has a high precision of contextual cues and as a result, it employs a global viewing strategy, (see Fig. 10A). However, the model in Fig. 9B has a low precision of contextual cues. This model believes that the contextual cues are non-informative and as a result, the agent employs a local viewing strategy, namely a decreased attention to the faces that define the social context (see Fig. 10B).

One can stratify these agents in terms of their prior beliefs, using their employed viewing strategies in Fig. 10. In the first simulation (Fig. 10A), the agent is unable to lower the precision of ambiguously happy faces under a sad context and vice versa. This agent is described in terms of the likelihood matrix of the generative model shown in Fig. 5A. This agent attends to both the target-face and the social cues suggesting that the precision of the local (i.e., target-face) and global cues (i.e., contextual cues) are in balance (see Fig. 9A); however, this agent is still unable to attribute a mental state to the target-face as it is unable to utilise the acquired contextual information. In the second simulation (see Fig. 10B), the fact that the agent does not attend to the social cues suggests that there is an imbalance between the precision of local and global cues (see Fig. 9B). These two agents shared the same likelihood matrices that are shown in Fig. 5A and only differed in terms of the likelihood matrices that are shown in Fig. 9. The likelihood matrices that are shown in Fig. 5A express the belief structures of agents that cannot utilise contextual information to attribute an emotion to the *target-face*. The likelihood matrices that are shown in Fig. 9 express the belief structures underlying global and local viewing strategies. The parameters that control the precision of likelihood matrices have the potential to disambiguate between different belief structures in clinical conditions such as schizophrenia.

## Discussion

In this work, we introduced a model that can change its perception under different contexts and provided a computational account of contextual perception under active inference. Being inspired by the *social context appreciation task*^1^, we introduced a task where a target-face is displayed in different social contexts. The target-face can express happy and sad emotions, either unambiguously or ambiguously. Crucially, the facial expressions of ambiguously happy/sad target-faces were identical. The objective was to identify the emotion of the target-face. There were two additional faces in the scene, other than the target-face. The emotions of these faces defined the social context. The social context could be either coherent (i.e., both faces express the same emotions) or incoherent (i.e., one face express happy the other sad emotion).

In this paradigm, contextual perception corresponds to attributing the same emotional state to the target-faces (with ambiguous emotions) as the emotion conveyed by the social context. An example is attributing a happy emotion to the target face because the social context conveys a happy emotion. Computationally, this can be expressed by down-weighting the precision of the mapping from *happy* context hidden state to the facial expressions of *ambiguously sad* faces. The same relation applies to *ambiguously happy* faces under a *sad* context. In other words, it allows us to reach an inference about ambiguous faces through ignoring features incongruent with that inference when the social context is in its favour.

Context congruency has been shown to improve performance in object identification tasks. These studies generally show that the objects are identified more accurately and faster when they are embedded in a congruent context^20–22^. Context congruence has been shown to improve reaction times in paradigms that involve threat of shock as well^23^. This study has shown that people identify fearful faces faster when there is a threat of shock than when there is no threat of shock (i.e., *safe*). Here, the congruence between the emotion of the face (i.e. *fearful*) and the context (i.e., *threat*) in which the face is observed improved the reaction times. In our paradigm, embedding the target-face in emotionally congruent contexts improved accuracy and decision times (see Fig. 8A).

Mismatch in stimuli has been shown to impair performance. An example of this is the Stroop task^24^. The Stroop task involves identifying the ink colour of a colour-word when the colour-word is written in the ink of either the same as the colour-word or another colour (e.g., the word ‘red’ written in red vs the word ‘red’ written in blue). The mismatch between the ink colour and the colour-word has a profound effect on behaviour, namely an increase in the time it takes to identify the ink colour of the word and a decrease in accuracy. This task involves two modalities that are competing to explain the ink colour of the word, namely the *colour-word* and the *ink colour* of the word. Correct identification of the colour only depends on the *ink colour* modality, but an inability to discounting the task-irrelevant *colour-word* modality gives inconsistent information about the ink colour and impairs performance. Computationally, this corresponds to an inability to decrease the precision of task-irrelevant modalities^15^. The Stroop effect has been replicated in paradigms involving affective stimuli as well^25^. In our paradigm, we compared the decision times when the target-face that consisted of incoherent facial expressions (i.e., ambiguous emotion) with when the target-face consisted of coherent facial expressions (i.e., unambiguous emotion). Consistent with the Stroop effect, the agent identified faces with coherent expressions faster (see Fig. 8B). The association between frontal lobe dysfunction and impairment at the Stroop task hints at the functional anatomy that might underwrite contextualisation of the sort outlined here. The involvement of the frontal cortices and their subcortical connections (e.g., to thalamus) is further endorsed by studies demonstrating the ability of these networks to differentially weight various sources of sensory data and their role in attention. In a mice study, the mediodorsal thalamus (MD) has been shown to sustain representations of task rules in the prefrontal cortex by amplifying local prefrontal cortex connectivity^26^. Another study revealed the role of the thalamic reticular nucleus (TRN) in attention in a 2-AFC task, where the mice were supposed to select between task-relevant (either auditory or visual) stimuli. Optogenetic manipulation of visual parts of the TRN caused a decline in the appropriate selection of stimuli^27^. These results highlight the role of thalamic nuclei in context-sensitive gain control. The role of the thalamus in these processes is not restricted to mouse research. A primate study involving pulvinar inactivation showed increased gaze shift to the ipsilesional hemifield, even though the saccades to the contralesional hemifield were intact in a visual task where targets can appear both ipsa- and contra- lesional hemifield^28^. Taken together, there is enough evidence that indicates that thalamus does more than merely relay information to the cortex, and that several thalamic nuclei are involved in attentional processes^29,30^. Abnormalities in fronto-thalamic networks, therefore, might contribute to attentional deficits in a way that prevents contextualisation of sensory input.

These neurobiological observations, concerning gain control, are central in theories of psychotic disorders. Specifically, the dysconnection hypothesis suggests that schizophrenia is due to aberrant synaptic gain control, especially in the circuits involving the prefrontal cortex^31^. A meta-analysis of diffusion tensor imaging studies suggests that white matter tracts connecting various regions such as frontal lobe and thalamus might indeed be affected^32^. Impairments in gain control processes can underly some of the positive symptoms of schizophrenia, such as delusions and hallucinations, in terms of an improper weighting of sensory evidence relative to prior beliefs about the causes of the sensory inputs^31^. In our paradigm, faces that express ambiguous emotions may be contextualised through attentional modulation. We suggest that this mechanism may be implemented through thalamus-dependent prefrontal gain-control. Abnormalities in these networks might explain why patients with schizophrenia differ from controls in terms of the perceived affective state in the *social context appreciation task*^1^.

There are studies that point out to abnormalities in gain control on tasks involving lower-level visual processing in schizophrenia as well. Dakin and colleagues showed that when a textured disk (target object) is embedded in a high contrast background, the controls are more likely to report it lower contrast than it is, while patients with schizophrenia reported the contrast levels more accurately^9^. Patients with schizophrenia seemed to be less prone to this contrast-contrast illusion, as well as to other visual illusions^33,34^. One potential mechanism that might cause the target object to appear lower contrast is lateral inhibition^35^. Under the lateral inhibition account, the neurons that respond to high contrast background suppress the neurons that respond to the lower contrast target object. Butler and colleagues suggest that there might be a reduced center-surround antagonism and contrast gain control in schizophrenia^8^. A rodent study showed that TRN neurons might be involved in lateral inhibition in the thalamus^36^. A review of TRN in the context of schizophrenia point out to how TRN might be involved in lateral inhibition through a cortex-TRN-thalamus circuit^37^. TRN is known to be GABAergic^37^, and impairments in GABAergic inhibition may underwrite some of the differences in visual processing between controls and patients with schizophrenia^38^.

The dopamine hypothesis of schizophrenia suggests that dysregulation of the dopaminergic system is responsible for some of the positive symptoms of schizophrenia. One of the findings that support this hypothesis is that antipsychotic drugs are mainly dopamine antagonists^39^. Kapur and colleagues proposed that the dysregulated dopaminergic system leads to aberrant attribution of salience to stimuli^40,41^. Some suggested that the process of aberrant salience is similar to a loss of signal to noise ratio, where phasic dopamine responses to salient stimuli are overwhelmed by increased noise in the system (e.g. tonic responses)^42^. This might, in turn, cause difficulties in learning stimuli-reinforcement associations^43^ and contribute to attentional deficits when the stimuli-reinforcement associations need to be utilised.

The dopaminergic midbrain (VTA/SN) and its projections to the striatum have been hypothesised to encode the precision of beliefs about future actions (or policies)^44^. The precision of future actions is expressed in terms of an inverse temperature parameter of a softmax function in the MDP formulation of active inference. Learning environmental contingencies (i.e., structure learning) in terms of Dirichlet parameter accumulation depends on the beliefs about the policies and thus also indirectly depend on this precision term^45^. This means that the variables representing dopaminergic signalling in the MDP model have direct consequences on learning and their impairment will thus disrupt attentional modulation, or the selective amplification of different sensory channels in silico. Although we did not consider a structure learning problem in this study, it would be fairly straightforward to incorporate it, as shown elsewhere (see^45^).

Kapur^40^ explains that contextually-guided salience attribution is impaired under the aberrant salience hypothesis. This may lead to abnormal beliefs about the internal states that represent the external world. Under the active inference framework, these beliefs depend on precision-weighted sensory observations. Contextual perception is a result of up-weighting context-relevant sensory data (or down-weighting context-irrelevant sensory data). In other words, contextual perception is a result of modulating sensory channels (via precision terms) that provide relevant information, and this process is identical to gain control account of attention. Abnormal precision encoding can thus lead to the aberrant assignment of importance to irrelevant stimuli. This might be one of the reasons why people with schizophrenia with delusions view face areas without distinguishing features more than controls^46^.

Intranasally administered oxytocin has been shown to increase fixations on the eye-region when healthy males view human faces^47^. A recent study showed that oxytocin not only improves exploratory viewing (e.g., in terms of the number of fixations, dispersion, etc.) in schizophrenia in response to images of facial stimuli but also images of inanimate objects^7^. In our work, the agent does not attend to the social cues that are necessary to infer the social context due to an imbalance between the precisions of local and global cues (see Fig. 10B). Reduced exploratory viewing is consistently reported in visual search studies of schizophrenia^5,48^. Could oxytocin alleviate the severity of this imbalance and allow for social cues to shape perception? An improvement in emotion recognition might be due to enhanced exploratory viewing, which has the potential explain why oxytocin seem to have positive effects on social-behavioural tasks^49^.

In the *social context appreciation task*^1^, the fixation duration of people with schizophrenia on contextual information was shorter and their perception of the mental state of target characters was less accurate than that of healthy participants when the target character was embedded in a social context. In our paradigm, two separate prior beliefs can explain these behaviours: (i) Decreased fixation duration on contextual information and decreased accuracy can be explained by an imbalance between the precisions associated with the social cues and target-character (i.e., ${z}_{sc}\approx 0$ and ${z}_{tf}\to \infty$, see “Materials and methods”). In active inference an action is more likely if it resolves uncertainty about the state of the world. With a low precision, the agent would not be able to resolve uncertainty about the social context and thus would not attend to the social cues. (ii) Decreased accuracy about the mental state of target characters can be explained by an inability to contextualise target-face emotion even when the social context is inferred correctly. This sort of contextualisation is described in terms of a low precision parameter $(\mathrm{i}.\mathrm{e}.,\mathrm{ z }\approx 0)$, see Figs. 5B and 6B. The agent cannot utilise contextual information to attribute a mental state to the target-character with ambiguous emotion when the precision parameter is high (i.e., *z* → ∞), see Figs. 5A and 6A. These behavioural responses might be due to an inability to selectively up or down weight sensory precisions to acquire reliable information^4,50^. Studying individual differences in terms of prior beliefs has the prospect of disambiguating the distinct causes of abnormal behaviours^51^.

This work has some limitations. In our model, we considered a very narrow space of facial expressions and contexts. The facial features that we consider are merely a subset of those that might be present when expressing these emotions. It has been suggested that facial recognition is based on more holistic processing than parts-based processing^52–54^. Configural information seems to be more influential than feature-based information in emotion recognition studies as well^54^. That being said, the study by Chen & Chen supports the idea that there are limited interactions between facial features in the upper and lower parts of the face in an emotion judgement paradigm when happy and sad facial expressions are presented in the fovea^55^. In this paradigm, the expressions of faces were modulated to create a spectrum between sadness and happiness. The upper and lower halves of happy and sad faces were displayed in aligned and misaligned face conditions. In the misaligned condition, the upper and lower halves of the faces were shifted laterally. The authors argue that facial expression classification should be different between aligned and misaligned conditions under a holistic processing view. This study showed that the classification of aligned and misaligned faces did not differ, thus supporting the idea that the local facial features are crucial in facial expression classification. The same study also showed that modulating the level of happy and sad expressions in the lower face influenced expression classification to a greater extent than the upper face. Another study showed that applying noise to the lower half of the face profoundly influenced facial expression classification^56^. The perceived eye expression differed when the expression in the lower half of the face was altered with noise. These results support the idea that some facial features might be more influential (i.e., salient) in facial expression classification. In our model, the facial expressions of faces that define the social context were equally informative about the emotion. This means that sampling either the eye or the mouth location was sufficient to infer the emotion of these faces. This is because the faces that define the social context always express an unambiguous emotion (i.e., the emotion expressed in the eye and mouth areas are coherent). The target face could express either an ambiguous or unambiguous emotion. This means that sampling both the eye and the mouth locations are necessary to infer target face emotion. In our model, one can change how informative the facial features in the upper and lower halves of the face are by modulating the precision parameters associated with mouth and eye locations. The agent would be less likely to sample the eye locations if the precision parameters associated with these locations are reduced. One can use the visual choices of the participants in an eye-tracking study and estimate the precision parameters associated with the lower and upper halves of the faces. This can reveal which locations are more informative in a paradigm like the one we considered here.

In this work, describing emotions in terms of changes in a set of isolated features was a simplification of how affective states might manifest (across the whole image) and considering facial configurations or interactions between facial expressions relevant to emotions would be a more accurate way of capturing differences between emotions. Moreover, people might be using different facial configurations when expressing similar emotions, and there is no one-to-one mapping between facial configurations and affective states^57^.

Although some of the features we used are commonly observed in happy/sad faces, we acknowledge that these features can be found in other emotions, as well as when a combination of emotions gives rise to compound facial expressions^58^. For example, it is plausible to have covered teeth when sad and angry emotions are elicited separately and when a combination of the two is elicited together. Suppose we expand the state space to include other emotions such as anger. In that case, the agent will require extra information to disambiguate between sad and angry faces, given that it observes covered teeth. There are two ways to improve the inference over emotional states. Firstly, the agent can fixate to a face location that is more informative about the emotions. Secondly, we can add different features to consider the differences between sad and angry emotions (e.g., covered teeth with lowered lip corners, covered teeth with tightly shut lips^16,59^). Although we considered a very limited space of facial expressions in our model, the model may be extended to facilitate greater complexity in the mapping between (combinations of) facial features and emotional categories. To do so one may link the MDP with a pre-trained (deep) generative model of facial expressions^60^, allowing for inference on emotional category by way of inference on the latent variables of this deep generative model. This extension would allow for this paradigm to be scaled, and for more complex emotions to be considered, but is beyond the scope of the simulations considered in this manuscript.

In our model, we expressed the social context in terms of a hidden state that the agent infers by sampling the features of the faces other than the target face. A more realistic way to represent the social context would be to take into account the temporally dependent interactions between context-specific visual cues. This would capture how events unfold over time and give rise to particular contexts. Defining a context in this way—within the sensory stream itself—is an alternative to our approach and may afford greater ecological validity to the study of context and social context specifically in future work. We assumed that a happy social context causes an ambiguous target-face to be perceived as happy (and a sad social context causes an ambiguous target-face to be perceived as sad), however, there are many real-life scenarios where the happy emotion conveyed by the social context does not warrant the happiness of the target character. In future work, we hope to scale these models up to the high dimensional visual data associated with emotional inference in ecologically valid settings.

## Materials and methods

### Active inference

The natural exchange of biological systems with their environment requires them to recognise any environmental changes and possibly take action to keep themselves within the physical boundaries that support their existence^61^. Mathematically, obeying such boundaries can be expressed by minimising the Shannon entropy^62^ of observable outcomes:

3 $$H\left[ {P\left( {\tilde{o}|m} \right)} \right] = - E_{{P\left( {\tilde{o}|m} \right)}} \left[ {\ln P\left( {\tilde{o}|m} \right)} \right]$$

Here, $\tilde{o }$ is the sequence of observations over time ($\tilde{o }=[{o}_{1},{o}_{2},\dots ,{o}_{T}])$, $m$ is the model under which model evidence $P(\tilde{o }|m)$ is evaluated, and $H$ and $E$ correspond to entropy and expectation, respectively.

Model evidence $P(\tilde{o }|m)$ can be expressed as the marginal of a joint probability distribution,

4 $$P\left( {\tilde{o}|m} \right) = \sum\limits_{x} {P\left( {\tilde{o},x|m} \right)}$$

In Eq. (4), $x$ represents the hidden causes. Model evidence $P(\tilde{o }|m)$ cannot be evaluated directly as the summation above is often intractable. Instead, we use a proxy on surprise $-\ln P(\tilde{o}\, |\, m)$, namely variational free energy. Variational free energy^63^ is obtained by applying Jensen’s inequality (for concave functions) to surprise:

5 $$\begin{aligned} - \ln P\left( {\tilde{o}|m} \right) & = - \ln \sum\limits_{x} {P\left( {\tilde{o},x|m} \right)} \\ & = - \ln \sum\limits_{x} {Q\left( x \right)\frac{{P\left( {\tilde{o},x|m} \right)}}{Q\left( x \right)}} \le \underbrace {{ - \sum\limits_{x} {Q\left( x \right)\ln \frac{{P\left( {\tilde{o},x|m} \right)}}{Q\left( x \right)}} }}_{Variational\,\,Free\;\;Energy} \\ \end{aligned}$$

Here, the right-hand side of the inequality is the variational free energy and $Q\left(x\right)$ is the approximate posterior beliefs over hidden causes $x$. The free energy can be rearranged in different ways to emphasise its properties,

6 $$F = - E_{Q\left( x \right)} \left[ {\ln P\left( {\tilde{o},x|m} \right)} \right] - H\left[ {Q\left( x \right)} \right]$$

7 $$\quad = - \ln P\left( {\tilde{o}|m} \right) + D_{KL} \left[ {Q\left( x \right)||P\left( {x|\tilde{o},m} \right)} \right]$$

8 $$\quad = \underbrace {{ - E_{Q\left( x \right)} \left[ {\ln P\left( {\tilde{o}|x,m} \right)} \right]}}_{Accuracy} + \underbrace {{D_{KL} \left[ {Q\left( x \right)||P\left( {x|m} \right)} \right]}}_{Complexity}$$

The notation ${D}_{KL}\left[Q||P\right]$ indicates the dissimilarity between the distributions $Q$ and $P$ in terms of KL-divergence^64^—i.e., a relative entropy. Variational free energy is a functional of an approximate posterior distribution over the hidden causes $Q\left(x\right)$ and the generative model $P\left(\tilde{o },x|m\right)$, see Eq. (6). The generative model describes the agent’s beliefs about the real-world dynamics in terms of likelihood mappings, state transition matrices and prior beliefs. Equation (7) ensures that free energy will always be greater than (or equal to) surprise due to the nonnegativity of the KL divergence, providing an upper-bound on surprise. Minimising free energy minimises the divergence between $Q\left(x\right)$ and $P(x|\tilde{o },m)$, making $Q\left(x\right)$ an approximation of the true posterior distribution $P(x|\tilde{o },m)$. Optimising the beliefs about hidden causes $Q\left(x\right)$ with respect to the free energy, minimises the divergence between surprise $-\mathrm{ln}P\left(\tilde{o }|m\right)$ and free energy $F$, making free energy an approximation to the surprise (or negative log model-evidence). Free energy can also be expressed in terms of accuracy and complexity, see Eq. (8). Accuracy is a measure of how accurately the observed outcomes can be explained under the current posterior beliefs $Q\left(x\right)$ whereas complexity measures the dissimilarity between posterior beliefs $Q\left(x\right)$ and prior beliefs $P\left(x|m\right)$.

In this work, we apply the active inference formalism to partially observable Markov decision processes (MDP).

### MDP generative model

**Figure 11:**
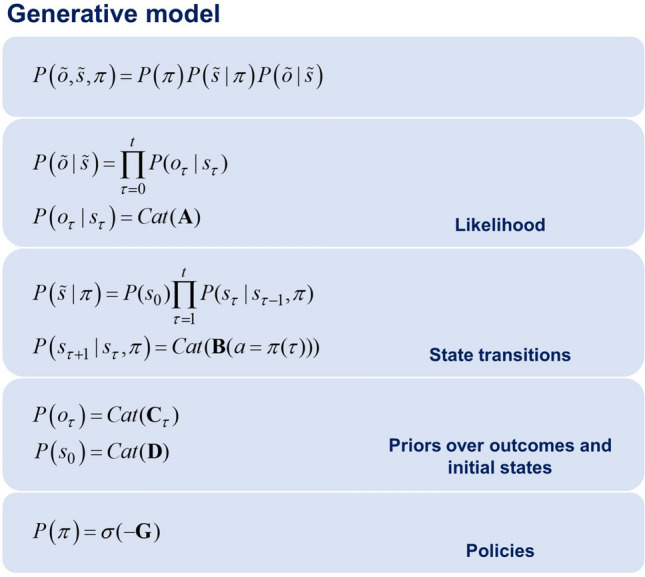
Markovian generative model. The generative model is expressed in terms of a joint probability distribution over the outcomes ($\tilde{o }$), hidden states ($\tilde{s }$) and policies ($\pi$). Here, $\tilde{o }$ and $\tilde{s }$ represent the sequence of observations and hidden states over time, respectively. The generative model captures the joint probability of three distributions (i) a mapping from hidden states $s$ to outcomes $o$ (expressed by the likelihood matrix $A$), (ii) a mapping from hidden states in the current time step ${s}_{\tau }$ to the next ${s}_{\tau +1}$ as a function of actions ($a$), where actions are sampled from the beliefs about the policies and (iii) prior beliefs over the policies ($\pi$), which is expressed in terms of a softmax function of (negative) expected free energy ($G$). Prior preference matrix ($C$) expresses the prior beliefs over outcomes. Initial state probability vector ($D$) expresses the beliefs about states at the initial time step $P\left({s}_{0}\right)$. The notation $Cat$ denotes categorical distribution. See the text for details.

The generative model is formulated as an MDP that describes the model’s beliefs about the world in terms of probability distributions over the hidden states and outcomes. The likelihood matrix (${\varvec{A}}$) describes how likely an outcome is, given the hidden states. The transition matrix (${\varvec{B}}$) comprises probabilistic mappings from the hidden states in the current time step to the hidden states in the next time step. The generative model includes prior beliefs about the initial hidden states (${\varvec{D}}$) and outcomes (${\varvec{C}}$). See Fig. 11 for the Markovian generative model.

We now draw a distinction between two sorts of hidden cause $x$ that were lumped together in Eq. (6). These are hidden states $s$ and policies $\pi$. We refer to the states of the environment that we cannot directly observe as ‘hidden’. Hidden states are the latent aspects of the world that give rise to observations and must be inferred. A policy is a sequence of actions, or control states, that the model can pursue.

9 $$\begin{aligned} F & = - E_{{Q(\tilde{s},\pi )}} \left[ {\ln P\left( {\tilde{o},\tilde{s},\pi |m} \right)} \right] - H\left[ {Q\left( {\tilde{s},\pi } \right)} \right] \\ & = E_{Q\left( \pi \right)} \left[ { - E_{{Q(\tilde{s}|\pi )}} \left[ {\ln P\left( {\tilde{o},\tilde{s}|\pi } \right)} \right] - H\left[ {Q\left( {\tilde{s}|\pi } \right)} \right]} \right] + D_{KL} \left[ {Q\left( \pi \right)||P\left( \pi \right)} \right] \\ & = E_{Q\left( \pi \right)} \left[ {F_{\pi } } \right] + D_{KL} \left[ {Q\left( \pi \right)||P\left( \pi \right)} \right] \\ \end{aligned}$$

We drop the conditioning on the model in the second line, but this should be assumed to be implicit throughout. Free energy depends on three terms, (i) ${F}_{\pi }$ is the free energy under a given policy, (ii) $Q\left(\pi \right)$ is the posterior beliefs about the policies and (iii) $P\left(\pi \right)$ is the prior beliefs about the policies.

${F}_{\pi }$ may be decomposed into a series of free energies for each time-step,

10 $$F_{\pi } = \sum\limits_{\tau } {F_{\pi \tau } }$$

11 $$F_{\pi \tau } = - E_{{Q\left( {s_{\tau } |\pi } \right)Q\left( {s_{\tau - 1} |\pi } \right)}} \left[ {\left[ {\tau \le t} \right] \cdot \ln P\left( {o_{\tau } |s_{\tau } } \right) + \ln P\left( {s_{\tau } |s_{\tau - 1} ,\pi } \right) - \ln Q\left( {s_{\tau } |\pi } \right)} \right]$$

The term $\left[\tau \le t\right]$ returns ‘1’ only if the condition inside the bracket is true (i.e., the present or past); otherwise (i.e., the future), it returns zero. This accounts for the fact that observations that have yet to be obtained are, by definition, not available. Under active inference, prior beliefs about policies $P\left(\pi \right)$ are expressed in terms of the expected free energy ${G}_{\pi }$. Minimizing this quantity ensures that the agent will also minimize its surprise. Setting the log probability of policies inversely proportional to the expected free energy ensures that the policies with the least expected free energy are more likely to be pursued $\mathrm{ln}P\left(\pi \right)\propto -{G}_{\pi }$.

Upon observing an outcome, the MDP model first optimises its beliefs about the hidden states and uses these beliefs to make predictions about the future. Crucially, the model can control the transitions of certain states as a function of action. An action is sampled from the posterior beliefs about policies at each unit time, where the posterior beliefs about the policies depend on their negative expected free energies. The expected free energy is

12 $$G_{\pi } = \sum\limits_{\tau } {G_{\pi \tau } }$$

13 $$G_{\pi \tau } = - \underbrace {{E_{{Q\left( {o_{\tau } |\pi } \right)}} \left[ {D_{KL} \left[ {Q\left( {s_{\tau } |o_{\tau } ,\pi } \right)||Q\left( {s_{\tau } |\pi } \right)} \right]} \right]}}_{Epistemic\,\,value} - \underbrace {{E_{{Q\left( {o_{\tau } |\pi } \right)}} \left[ {\ln P\left( {o_{\tau } } \right)} \right]}}_{Extrinsic\,\,value}$$

14 $$\quad = \underbrace {{D_{KL} \left[ {Q\left( {o_{\tau } |\pi } \right)||P\left( {o_{\tau } } \right)} \right]}}_{Risk} + \underbrace {{E_{{Q\left( {s_{\tau } |\pi } \right)}} \left[ {H\left[ {P\left( {o_{\tau } |s_{\tau } } \right)} \right]} \right]}}_{Ambiguity}$$

Here, $Q\left({o}_{\tau },{s}_{\tau }|\pi \right)=P\left({o}_{\tau }|{s}_{\tau }\right)Q\left({s}_{\tau }|\pi \right) \approx P\left({o}_{\tau },{s}_{\tau }|\tilde{o },\pi \right)$. Expected free energy ${G}_{\pi }$ is obtained by summing ${G}_{\pi \tau }$ over future time steps $\tau >t$, see Eq. (12). Expected free energy can be expressed in terms of epistemic and extrinsic values, see Eq. (13). Epistemic value is the Bayesian surprise^65^ expected under predicted outcomes for a given policy. It expresses how much the beliefs about hidden states diverge from the beliefs about hidden states when predicted outcomes under a policy are taken into account^66,67^. Extrinsic value is the expected log probability of outcomes under a policy^68^. Expected free energy can also be expressed in terms of risk and ambiguity^69^, see Eq. (14). Risk is expressed as a KL divergence between predicted outcomes and outcomes preferred a priori. The closer the predicted outcomes (under a policy) are to the preferred outcomes, the more likely that policy will be pursued. Ambiguity is an expected entropy over the likelihood term. This term expresses that a policy is more likely to be pursued if it leads to more certain outcomes.

### Variational updates

#### Perception

The equations below summarise perceptual inference as the optimisation of posterior beliefs about hidden states with respect to free energy under a given policy ${F}_{\pi }$:

15 $${\varvec{s}}_{\tau }^{{\pi^{*} }} = \sigma \left( {\ln {\varvec{B}}_{\tau - 1}^{\pi } {\varvec{s}}_{\tau - 1}^{\pi } + \ln {\varvec{B}}_{\tau }^{\pi } \cdot {\varvec{s}}_{\tau + 1}^{\pi } + \zeta \ln {\varvec{A}} \cdot o_{\tau } } \right)$$

16 $${\varvec{\varepsilon}}_{\tau }^{\pi } = \ln {\varvec{s}}_{\tau }^{{\pi^{*} }} - \ln {\varvec{s}}_{\tau }^{\pi ,current}$$

17 $${\varvec{s}}_{\tau }^{\pi ,new} = \sigma \left( {\ln {\varvec{s}}_{\tau }^{\pi ,current} + \lambda {\varvec{\varepsilon}}_{\tau }^{\pi } } \right)$$

Here, ${{\varvec{s}}}_{\tau }^{\pi }$ corresponds to the posterior beliefs about hidden states under a policy $Q\left({s}_{\tau }^{\pi }|\pi \right)$. The first equation above shows the optimal solution ${{\varvec{s}}}_{\tau }^{\pi *}$ to the state estimation problem, see Eq. (15). The difference between (log) optimal solution and (log) current beliefs about the hidden states generates a state prediction error see Eq. (16), where ${{\varvec{\varepsilon}}}_{\tau }^{\pi }=-\frac{\partial {F}_{\pi }}{\partial {{\varvec{s}}}_{\tau }^{\pi }}$. Finally, the beliefs about the hidden states are updated via each iteration of a gradient descent algorithm, see Eq. (17). Here $\sigma$ is a softmax function—ensuring ***s*** is confined to a simplex^70^ (where its elements sum to one)—and $\lambda$ is the learning rate. Equations (16) and (17) are repeated until the state prediction error is suppressed ${{\varvec{\varepsilon}}}_{\tau }^{\pi }\approx 0$.

#### Attention

The $\zeta$ in Eq. (15) is a precision (or inverse temperature) parameter applied to the likelihood matrix ${\varvec{A}}$. This parameter manipulates the precision of the mapping from hidden states to outcomes^19^. For each hidden state there is a precision term

18 $$P\left( {o_{\tau } = j|s_{\tau } = i,\zeta_{i} } \right) = \frac{{{\varvec{A}}_{ji}^{{\zeta_{i} }} }}{{\sum\limits_{k} {{\varvec{A}}_{ki}^{{\zeta_{i} }} } }}$$

Here $i\in I$ and $j\in J$ where *I* is the space of hidden states in a hidden state dimension and *J* is the space of outcomes in a modality. Under active inference, attention corresponds to inferring the precision of sensory observations and their hidden causes^14^. This (Gibbs) parameterisation has been widely exploited to simulate phenomena from neuronal synchronisation^71^ through to visual illusions^72^. In the current work, we use this parameter to demonstrate how contextual perception can occur under active inference.

#### Policy evaluation

In the policy evaluation phase, the model evaluates the policies in terms of their free energy under a given policy ${{\varvec{F}}}_{\pi }$ and expected free energy ${{\varvec{G}}}_{\pi }$. A policy is more likely if it minimises both ${{\varvec{F}}}_{\pi }$ and ${{\varvec{G}}}_{\pi }$.

19 $${\varvec{\pi}} = \sigma \left( { - {\varvec{F}}_{\pi } - {\varvec{G}}_{\pi } } \right)$$

where ${\varvec{\pi}}$ is the posterior beliefs about the policies $Q\left(\pi \right)$, and ${F}_{\pi }$ and ${G}_{\pi }$ are expressed as

20 $${\varvec{F}}_{\pi } = -\sum\limits_{\tau } {{\varvec{\varepsilon}}_{\tau }^{\pi } } \cdot {\varvec{s}}_{\tau }^{\pi }$$

21 $${\varvec{G}}_{\pi } = \sum\limits_{\tau } {\left( {{\varvec{o}}_{\tau }^{\pi } \cdot \left( {\ln {\varvec{o}}_{\tau }^{\pi } - {\varvec{C}}_{\tau } } \right) + {\varvec{H}} \cdot {\varvec{s}}_{\tau }^{\pi } } \right)}$$

Here, ${{\varvec{o}}}_{\tau }^{\pi }$ are the expected outcomes under a policy $Q\left({o}_{\tau }^{\pi }|\pi \right)$ at a future time step τ. ${{\varvec{C}}}_{\tau }$ is the outcomes that the model expects a priori and ${\varvec{H}}$ is the entropy of the outcomes for all possible combinations of hidden states ${\mathbf{H}} = - E_{{P\left( {o_{\tau } |s_{\tau } } \right)}} \left[ {\ln P\left( {o_{\tau } |s_{\tau } } \right)} \right]$.

#### Action selection (and Bayesian model averaging)

In the action selection phase, an action ${a}_{t}$ is sampled from the policies.

22 $$a_{t} = \max_{a} {\varvec{\pi}}$$

The chosen action is the one that is most likely to fulfil the expected outcomes. The model uses the expected hidden states to make predictions about the outcomes expected in the future.

23 $${\varvec{s}}_{\tau } = \sum\limits_{\pi } {{\varvec{\pi}}_{\pi } \cdot {\varvec{s}}_{\tau }^{\pi } }$$

24 $${\varvec{o}}_{\tau } = {\varvec{As}}_{\tau }$$

Beliefs about hidden states ${{\varvec{s}}}_{\tau }$ are obtained by weighing the beliefs about the hidden states expected under a policy ${{\varvec{s}}}_{\tau }^{\pi }$ with the probability of policies ${{\varvec{\pi}}}_{\pi }$. This corresponds to Bayesian model averaged beliefs about hidden states, where each policy is a model, see Eq. (23). Here, the bold **π** corresponds to the posterior beliefs about the policies $Q\left(\pi \right)$ whereas π in normal type corresponds to a policy (or sequence of actions). Expected outcomes are obtained by weighing the beliefs about outcomes under each hidden state ${\varvec{A}}=P\left({o}_{\tau }|{s}_{\tau }\right)$ with the beliefs about hidden states ${{\varvec{s}}}_{\tau }$, see Eq. (24).

### Contextual perception

Here, we describe how contextual perception can occur under an MDP model of active inference, using the mental state attribution task. We assumed that the social context would influence the perceived emotion of emotionally ambiguous target-faces the most. This is because the facial features alone leave some uncertainty unresolved. For contextual perception to happen, we assumed the following three conditions:

(i) the target-face should express an ambiguous emotion,
(ii) the social context should provide coherent information (i.e. both faces that define the context express either happy or sad emotions),
(iii) The agent should be able to attribute the facial expressions of the target-face to an emotional state that is consistent with the social context.

An example is, identifying a target-face with ambiguous emotion as happy when the social context conveys a happy emotion (i.e. the other two faces are happy). This requires the agent to dissociate the facial expressions of the target-face with the sad emotion when the social context conveys a happy emotion. This can be expressed in the agent’s generative model in the following way: (i) under the *happy* context, the relative precision of the facial expressions of ambiguously sad faces is lower compared to the precision of ambiguously happy faces, (ii) under the *sad* context, the precision of the ambiguously happy faces is lower compared to the precision of ambiguously sad faces. We can express these mathematically with the equations below,

25 $$A_{nijkl}^{m} = P\left( {o^{m} = n|s^{1} = i,s^{2} = j,s^{3} = k,s^{4} = l} \right)$$

26 $$\overline{P}\left( {o^{m} = n|s^{1} = i,s^{2} = j,s^{3} = k,s^{4} = l} \right) = \sigma \left( {\zeta_{ijk}^{m} \ln \overline{A}_{nijkl}^{m} } \right)$$

The likelihood of the outcomes in the generative process is described by Eq. (25). This equation describes the probability of the n-th outcome under the m-th modality ${o}^{m}=n$ with $m\in M$ and $n\in N$, where $M$ is the space of outcome modalities (*facial expressions*, *where*, *feedback*) and N is the space of outcomes under a modality (e.g. the outcomes in the *feedback* modality are null, correct and incorrect). The probability of the outcome ${o}^{m}=n$ is expressed as a function of hidden states ${s}^{1}=i$,${s}^{2}=j$, ${s}^{3}=k$ and ${s}^{4}=l$ where the superscripts correspond to different hidden state dimensions and $i\in I$ (i.e., *context*) , $j\in J$ (i.e., *target face emotion*), $k\in K$ (i.e., *target face type*) and $l\in L$ (i.e., *where*). Here the *I*, *J*, *K* and *L* correspond to the space of hidden states in each dimension respectively (e.g., *J* corresponds to the *target face emotion* hidden state dimension underwhich the possible states are happy and sad). See Fig. 4B for the generative process likelihood matrices in the mental state attribution task.

Equation (26) describes the probability of the outcomes for the generative model, where $\sigma$ is the softmax function and ${\zeta }_{ijk}^{m}$ is a scalar value that corresponds to the precision of the likelihood mapping from the *i-th*, *j-th* and *k-th* hidden states to the *m-th* outcome modality. The probability of outcomes in the generative model is obtained by applying a softmax function to the product of the precision term ${\zeta }_{ijk}^{m}$ and the logarithm of the likelihood mapping. Here the bar notation is used (see $\overline{P }$ and $\overline{A }$) to indicate the likelihood mapping for the generative model.

We now introduce precision matrices. Each entry in these matrices shows the values of the precision terms ${\zeta }_{ijk}^{m}$ for a combination of hidden states and outcomes.27

The first matrix in Eq. (27) shows the precision terms when the *context* and *target face emotion* are both happy. Note that the rows indicate modalities, while the columns are levels of the *target-face type* state. Similarly, the second matrix shows the precision terms when the context is happy and the target-face is sad. In both matrices, all precisions are set high (i.e. $\infty$) except for the mapping from the *ambiguously sad* target-face to facial expressions in the second matrix. The precision of the likelihood mapping from *ambiguously sad* target-face to facial expressions is expressed by $z$. When the precision $z$ is high (i.e. $z\to \infty$), the agent is equally likely to attribute happy and sad emotions to target-faces with ambiguous emotion (see Fig. 5A). However, when the precision $z$ is set low (i.e. $z\approx 0$) the agent would no longer be able to associate the facial expressions of an *ambiguously sad* target-face with the sad emotion under a happy context (see Fig. 5B left panel). The agent can only associate an emotionally ambiguous target-face with the happy emotion.28

Similarly, the matrices in Eq. (28) shows the precision terms when the *context* is sad. Here, the precision of the mapping from *ambiguously happy* target-face to facial expressions (see the first matrix above) is expressed by $z$. When the precision $z$ is high (i.e. $z\to \infty$), the agent is equally likely to attribute happy and sad emotions to target-faces with ambiguous emotion. However, when the precision $z$ is low (i.e. $z\approx 0$) the agent can no longer associate the facial expressions of an *ambiguously happy* target-face with the happy emotion under a sad context (see Fig. 5B right panel). The agent can only associate an ambiguous target-face with the sad emotion. In the precision matrices, we used infinity signs ($\infty$) and the digit zero ($z\approx 0)$ to emphasise the relative difference between precisions.

### Global versus local viewing

Here, we will describe the computational mechanisms underlying global and local viewing strategies. Mathematically, these strategies can be described using the below precision matrix:29

The precision matrix shows the mapping from the hidden state *Where* to the outcome modalities *Facial expression*, *Feedback* and *Where*. The columns are associated with the locations in the scene. Locations 2 and 3 are the locations associated with the eyes and the mouth of the target-face, respectively. Locations 4 to 7 are associated with the eyes and mouths of the faces that define the social context. Here, ${z}_{tf}$ is the precision of the target-face facial expressions and ${z}_{sc}$ is the precision of the facial expressions of the faces that define the social context.

When the precision ${z}_{tf}$ and ${z}_{sc}$ are both high, the agent would employ a global viewing strategy (see Fig. 10A). When the precision ${z}_{tf}$ is high and ${z}_{sc}$ is low, the agent would employ a local viewing strategy, namely a decreased attention to the faces that define the social context (see Fig. 10B).

## Data Availability

The simulations in this paper were obtained by using a standard software routine, spm_MDP_VB_X.m. This code is available in the SPM software for Matlab: http://www.fil.ion.ucl.ac.uk/spm/.
